# NIR II‐Guided Photoactivatable Silencing Polyplex Boosts Cancer Immunotherapy

**DOI:** 10.1002/EXP.20240047

**Published:** 2025-05-30

**Authors:** Yuquan Zhang, Jie Wang, Tian Zhang, Dongsheng Tang, Haiyin Yang, Shuai Guo, Yuchuan Fan, Caixia Sun, Haihua Xiao, Yuanyu Huang, Yuhua Weng

**Affiliations:** ^1^ School of Life Science, School of Interdisciplinary Science, Aerospace Center Hospital, Key Laboratory of Molecular Medicine and Biotherapy, Key Laboratory of Medical Molecule Science and Pharmaceutics Engineering Beijing Institute of Technology Beijing China; ^2^ Beijing National Laboratory for Molecular Sciences, Laboratory of Polymer Physics and Chemistry Institute of Chemistry Chinese Academy of Sciences Beijing China; ^3^ University of Chinese Academy of Sciences Beijing China; ^4^ School of Chemistry Chemical Engineering and Biotechnology Nanyang Technological University Singapore Singapore; ^5^ School of Medical Engineering, School of Interdisciplinary Science, Affiliated Zhuhai People's Hospital Beijing Institute of Technology (BIT) Zhuhai China; ^6^ Advanced Technology Research Institute Beijing Institute of Technology (BIT) Jinan China

**Keywords:** cancer immunotherapy, immunogenic cell death, NIR II‐guided photodynamic therapy, PD‐L1, small interference RNA

## Abstract

Photodynamic therapy (PDT) triggers immunogenic cell death (ICD) within the tumor microenvironment, consequently enhancing tumor immunotherapy. However, the maximum absorption wavelengths of first and second‐generation PDT photosensitizers limit the penetration depth of therapeutics, resulting in insufficient anti‐tumor outcomes. This study reports a custom‐designed polymer, PTSQ, which exhibits significant absorption in the near‐infrared region (NIR) window and fluorescence emission spectra within the NIR II range, demonstrating excellent PDT efficiency. Additionally, PTSQ self‐assembles into nanomicelles, exhibiting outstanding siRNA delivery. To further enhance tumor immunotherapy, we introduce an immune checkpoint blockade strategy and prepared PTSQ/siPD‐L1 complexes. We present a novel approach to tumor treatment by combining NIR light‐activated PDT and ICD to enhance siPD‐L1 therapy. At the cellular level, PTSQ/siPD‐L1 complexes exhibit potent induction of ICD while concurrently suppressing PD‐L1 gene expression. In vivo, these complexes significantly impede the growth of CT26, 4T1, and patient‐derived xenograft (PDX) tumors. This effect is achieved by promoting in situ ICD, which reverses tumor environment and activates immune cells in tumors and spleens, including T cells, dendritic cells (DCs), and macrophages. Overall, this study offers insights for the development of NIR II‐guided cancer immunotherapy and underscores the efficacy of PDT in conjunction with checkpoint blockade for cancer treatment.

## Graphical abstract

- Due to limitation of penetration therapy with first and second‐generation photodynamic therapy (PDT) photosensitizers, an NIR II‐guided photoactivatable complex was proposed via the self‐assembly of the photosensitive polymer PTSQ and the electrostatic adsorption of siPD‐L1 to enhance cancer immunotherapy.
- The PTSQ/siRNA could trigger complex destructive effects by generating ROS with laser irradiation, facilitating endosomal escape of siPD‐L1 and immunogenic cell death.
- The spatiotemporal synergy between PDT and immune checkpoint blockade effectively overcome immunosuppression within tumor microenvironment, ultimately enhancing the efficacy of cancer immunotherapy.

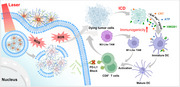

## 1 Introduction

Non‐invasive therapeutic approaches are paving the way for novel possibilities in cancer treatment. Photodynamic therapy (PDT), recognized as a common non‐invasive treatment, has found extensive application in clinical settings for treating various pathologies, including skin disorders, ophthalmic conditions, and cancers [1]. The fundamental components of PDT include a photosensitizer, light, and oxygen. When these three elements coexist, exposure to specific wavelengths of light activates the photosensitizer, prompting its interaction with oxygen in the tissue to generate toxic free radicals, inducing cytotoxic effects and leading to cell death [2, 3]. Within the PDT process, the photosensitizer plays a pivotal role, and an ideal photosensitizer should exhibit strong absorption within the near‐infrared window [4, 5, 6].

With an excitation wavelength below 650 nm, light undergoes uneven scattering in biological tissues and is absorbed by endogenous chromophores like hemoglobin, blood, melanin, and even lipids. The near‐infrared region (NIR) window (650–1700 nm) is particularly compatible with biological tissues, allowing for deeper tissue penetration and reduced photodamage, consequently yielding superior therapeutic outcomes [2]. Furthermore, compared to photosensitizers in the NIR I window (650–900 nm), those operating in the NIR II window (1000–1700 nm) have garnered significant attention due to their deeper tissue penetration abilities and minimized tissue scattering [7, 8].

It is well known that PDT induces immunogenic cell death (ICD), a distinctive form of cell demise that not only triggers immune responses but also holds the potential to enhance the activity and infiltration of cytotoxic lymphocytes (CTL) in vivo [9, 10]. ICD is characterized by the release of tumor‐associated antigens and damage‐associated molecular patterns (DAMPs), including surface‐exposed calreticulin (CRT), the release of high‐mobility group protein B1 (HMGB1), and the secretion of adenosine triphosphate (ATP) [11, 12, 13]. The exposure of CRT on the surface of dying cells, providing an “eat‐me” signal that recruits DCs, facilitating the engulfment of apoptotic cells or the presentation of tumor antigens, and promoting the maturation of DCs in the tumor microenvironment [14, 15]. Additionally, the extracellular release of ATP after cell damage/death serves as a crucial modulator of immune response under stressful conditions. This secreted ATP acts as a “Find me” signal, recruiting and activating immune cells such as T cells, neutrophils, and macrophages [16, 17]. Upon cellular injury or necrosis, nuclear HMGB1 is released into the extracellular milieu. This passively released HMGB1 can participate in ICD‐mediated immunogenesis, modulating immunostimulatory reactions by interacting with various Toll‐like receptors [18, 19], thereby activating macrophages and monocytes and eliciting cytokine‐mediated functions.

Despite the significant role of PDT in cancer immunotherapy, strategies involving immune checkpoint blockade, such as the use of PD‐1 and PD‐L1 antibodies, have garnered considerable attention and are increasingly considered frontline cancer drugs [20]. However, immune checkpoint blockade (ICB) is not universally effective for most patients, typically yielding positive responses in only 20%–30% of cases [21]. This is primarily due to the non‐immunogenic nature of the tumor microenvironment and the limited presence of tumor‐infiltrating T cells, hindering adaptive immune responses within the tumor [16, 22]. In contrast to antibodies, gene silencing technologies have emerged as potent tools in cancer immunotherapy [23, 24]. Through this approach, immune checkpoint genes in tumor cells can be effectively and specifically silenced. To date, the US Food and Drug Administration (FDA) has approved six small interfering RNA (siRNA) drugs, including Patisiran, Givosiran, Lumasiran, Inclisiran, Vutrisiran, and Nedosiran [25]. However, as naked siRNA cannot traverse cellular barriers to enter the cytoplasm, there is a consistent need for safe and efficient delivery systems to facilitate siRNA uptake into cells [26, 27]. Common siRNA delivery carriers include polymers, liposomes, peptides, and exosomes, among which polymers play a crucial role due to their flexible and controllable structures.

**SCHEME 1:**
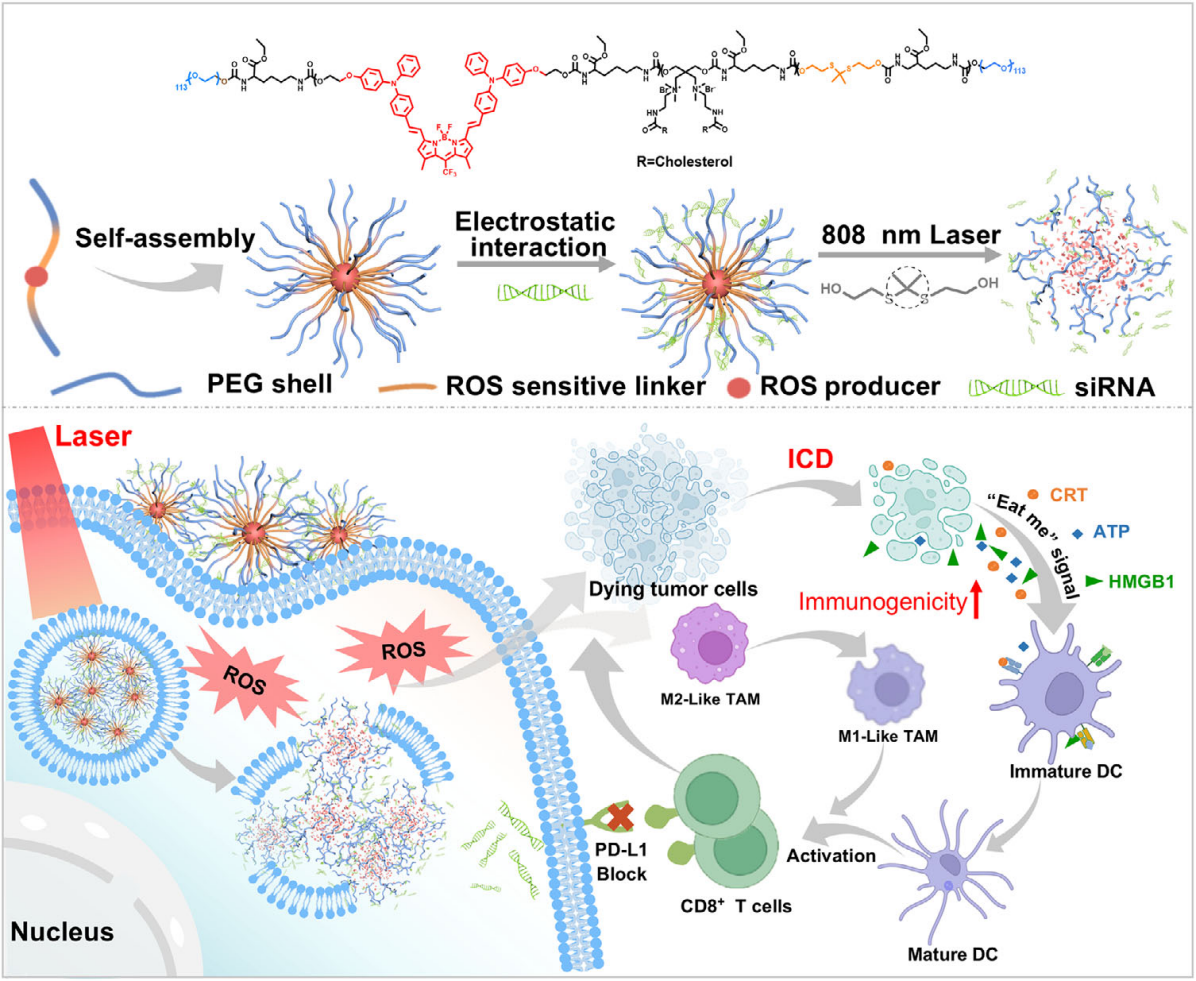
Illustration depicting NIR II‐guided PDT‐assisted checkpoint silencing therapy.

In this study, we developed an NIR II‐guided silencing photoactivatable polyplex to boost cancer immunotherapy. We initially synthesized a multifunctional photosensitive polymer, PTSQ, with absorption and emission capabilities in the NIR II window, efficiently generating ROS. Simultaneously, PTSQ could self‐assemble into nanomicelles and compact siRNA into the PTSQ/siRNA complex through electrostatic interactions. Under 808 nm laser irradiation, the ROS‐responsive linker in PTSQ broke the chain, leading to the disintegration of the complex, releasing the compacted siPD‐L1, and facilitating RNA lysosome escape. Furthermore, the ROS generated by the complex induced ICD, promoting the migration and maturation of DCs, thus initiating an anti‐cancer immune response. The siRNA used in the complex specifically silenced the expression of PD‐L1 in tumor cells, disrupted the PD1/PD‐L1 checkpoint pathway and enhanced CTL killing capability. Ultimately, an enhanced cancer immunotherapy was achieved by PDT‐assisted ICB therapy (Scheme 1).

## 2 Results and Discussion

### 2.1 Preparation and Characterization of NIR II Photoactivatable siRNA Complex

The PTSQ polymer was synthesized through condensation polymerization, as outlined in Figure S1. At first, the P1 polymer was formed by coupling the photosensitizer (M1), ROS‐cleavable linker 2'‐(propane‐2,2‐diylbis (sulfanediyl)) bis (ethan‐1‐ol) (DSB), 2,2‐bis(bromomethyl)‐1,3‐propanediol, and mPEG_5000_‐OH. Successful synthesis was confirmed by ^1^H NMR, as depicted in Figure S2. Subsequently, the P1 polymer was reacted with cholesterol derivative (M5) to yield the final PTSQ polymer. The identical polymer structures were confirmed through ^1^H NMR analysis (Figure S3).

**FIGURE 1:**
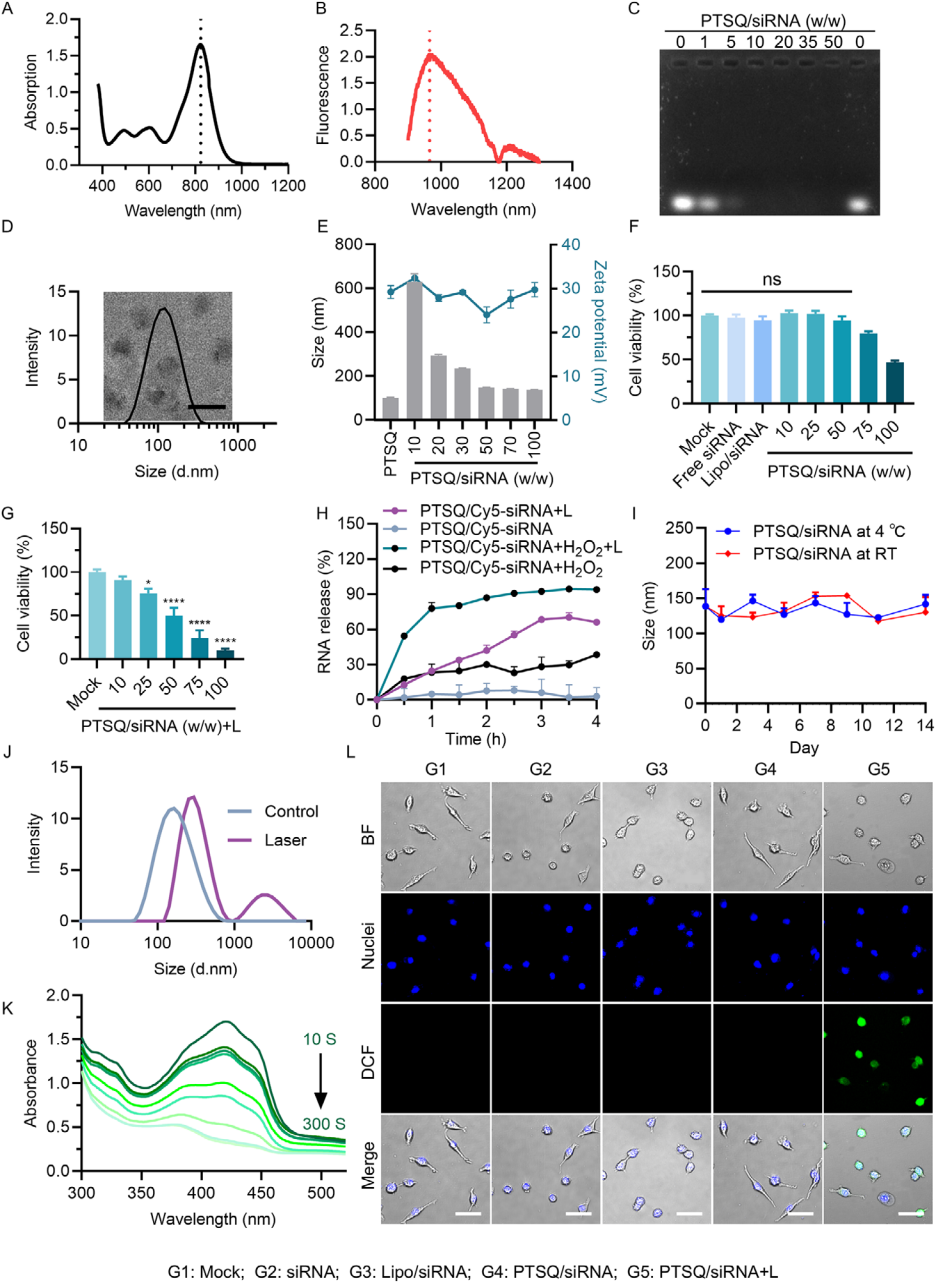
Characterization of PTSQ and PTSQ/siRNA complex. (A) Absorption and (B) fluorescence spectra of the PTSQ polymer. (C) Agarose gel retardation assay. (D) TEM image and particle size distribution of PTSQ/siRNA complex at PTSQ: siRNA mass ratios of 50:1. If not specified otherwise, the siRNA referred to in the manuscript denotes non‐sense siRNA (siNC). (E) Size distribution and zeta potential of PTSQ/siRNA complexes at various mass ratios. (F) Cell viability of CT26 cells after treatment with PTSQ/siRNA complex at various mass ratios in the absence or presence of laser irradiation (G), “L” represents 808 nm laser irradiation. (H) Cumulative in vitro release of siRNA from PTSQ/siRNA complex under 50 mM H_2_O_2_ or laser irradiation conditions. (I) Determination of the size of PTSQ/siRNA stored at room temperature and 4°C for an extended period. (J) Size changes of PTSQ/siRNA before and after laser irradiation. (K) The ability of PTSQ/siNC to produce ROS under various laser illumination times (0–300 s) was evaluated using the DPBF probe. (L) Cellular ROS production was assessed using the DCFH‐DA probe. Scale bar: 50 µm. The blue and green color corresponded to DAPI and DCFH‐DA staining, respectively. *p < 0.05, **p < 0.01, ***p < 0.001, ****p < 0.0001.

The primary drawback of common PDT lies in its limited tissue penetration. This restricted depth of penetration can compromise the effectiveness of cancer therapy, as the intensity of the excitation beam diminishes exponentially with increasing penetration depth. It is well‐established that the NIR window (650–1700 nm) is the most suitable range for interaction with biological tissues, facilitating deeper penetration. UV–vis absorption and fluorescence emission spectra of PTSQ were measured and presented in Figure 1A,B, respectively. The data revealed a robust UV peak with an absorption maximum at 823 nm, along with a broad emission signal spanning 900–1100 nm, with the most intense emission occurring at 966 nm. Consequently, PTSQ demonstrated NIR II emission capabilities, suggesting the potential for enhanced tissue penetration.

As is commonly understood, siRNA represents a type of biomacromolecule that cannot be readily taken up by cells [28, 29]. In order to facilitate synergistic PDT and gene silencing immunotherapy, the siRNA delivery capability of PTSQ was assessed by agarose gel retardation assay. As depicted in Figure 1C, an increase in the weight ratio between PTSQ and siRNA led to a corresponding rise in siRNA loading capacity. When this ratio reached 10 or above, PTSQ successfully loaded siRNA, forming the PTSQ/siRNA complex with no detectable free siRNA in the agarose gel. Subsequently, we examined the physical characteristics of the PTSQ/siRNA complex, including particle size, size distribution, and zeta potential. As shown in Figure 1D,E, the particle size and zeta potential of the PTSQ polyplex alone were approximately 100 nm and 30 mV, respectively. With an increase in the PTSQ: siRNA mass ratio from 10 to 50, the particle sizes gradually decreased to around 150 nm, reaching a plateau. The zeta potential exhibited slight fluctuations within the range of 20–30 mV. The slightly larger size of the complex at a ratio of 10 might be attributed to a relatively loose interaction between the positively charged polyplex and negatively charged nucleic acids.

Subsequently, we carried out the detection of the photo‐activation ability of the PTSQ/siRNA complex and its photodynamic therapy (PDT) effect on tumor cells. As can be seen from Figure 1F,G, when the mass ratio of the PTSQ/siRNA complex was below 50, there was almost no cytotoxicity; when the mass ratio increased to 100, a slight cytotoxicity appeared. However, the cells irradiated with the 808 nm laser show an obvious killing effect, and this killing effect was positively correlated with the mass ratio of the polymer to siRNA. Considering comprehensively the biocompatibility of the PTSQ/siRNA complex and the balance of the killing effect on tumor cells, we chose the mass ratio of 50 for the subsequent experiments.

We then conducted a systematic study on the release behavior of siRNA from the PTSQ/siRNA complexes. During the experiment, these complexes were placed in a PBS buffer and continuously stirred. Meanwhile, the laser irradiation group was irradiated with laser simultaneously during the stirring process. At the same time, considering the need to simulate the release situation of PTSQ/siRNA in the tumor cell microenvironment, we also investigated the release of PTSQ/siRNA in the hydrogen peroxide environment. After all, hydrogen peroxide is widely present in the tumor microenvironment.

It can be clearly seen from Figure 1H that, compared with the PTSQ/siRNA group, the release amounts of siRNA in the PTSQ/siRNA+L group (the laser irradiation group) and the PTSQ/siRNA+H_2_O_2_ group (the hydrogen peroxide environment group) were significantly increased. This indicated that both hydrogen peroxide and laser irradiation could promote the release of siRNA. More importantly, the PTSQ/siRNA+H_2_O_2_+L group (the group with both hydrogen peroxide environment and laser irradiation) was able to release up to 90% of siRNA within 4 h. This fully demonstrates that both laser irradiation and the reactive oxygen species in the tumor microenvironment can cause the disintegration of the PTSQ/siRNA complexes, thereby releasing siRNA.

Meanwhile, the long‐term stability of the system was also evaluated (Figure 1I). The dynamic light scattering (DLS) results indicated that the size of PTSQ/siRNA was very stable when stored at either room temperature (RT) or 4°C. Similarly, after 2 weeks of storage at RT, release of siRNA from the system was not observed (Figure S4). These findings led us to believe that the PTSQ/siRNA complex constituted a very stable drug system.

To study the photoresponsive character of PTSQ/siRNA, we examined the size changes before and after laser irradiation (808 nm, 4 min, 2 W cm^−2^) (Figure 1J). Notably, after laser irradiation, the PTSQ/siRNA complex underwent an increase in size, indicating potential destruction and collapse of the complex. To explore whether the cytotoxicity of the PTSQ/siRNA complex was predominantly attributed to the generation of reactive oxygen species (ROS), we utilized 1,3‐diphenylisobenzofuran (DPBF), a probe which can detect various ROS types, including O_2_^−^, H_2_O_2_, ·OH, and ^1^O_2_ [30]. The UV–vis absorption spectrum of the mixture was monitored at different time points after 808 nm irradiation, revealing a significant decrease in the intensity of DPBF absorption peak at 420 nm (Figure 1K). This suggested the quenching of the probe by the ROS generated from 808 nm light‐activated PTSQ/siRNA complex. Furthermore, ROS production was assessed with an oxidation‐sensitive fluorescent probe DCFH‐DA through confocal laser scanning microscopy (CLSM). The PTSQ/siRNA complex exhibited bright green fluorescence upon 808 nm laser irradiation, while no obvious fluorescence was observed in the PTSQ/siRNA complex group without laser irradiation (Figure 1L). These results indicated that both PTSQ and PTSQ/siRNA complex exhibited excellent NIR II emission and in vitro PDT effects.

### 2.2 In Vitro Gene Silencing and Lysosomal Escape

**FIGURE 2:**
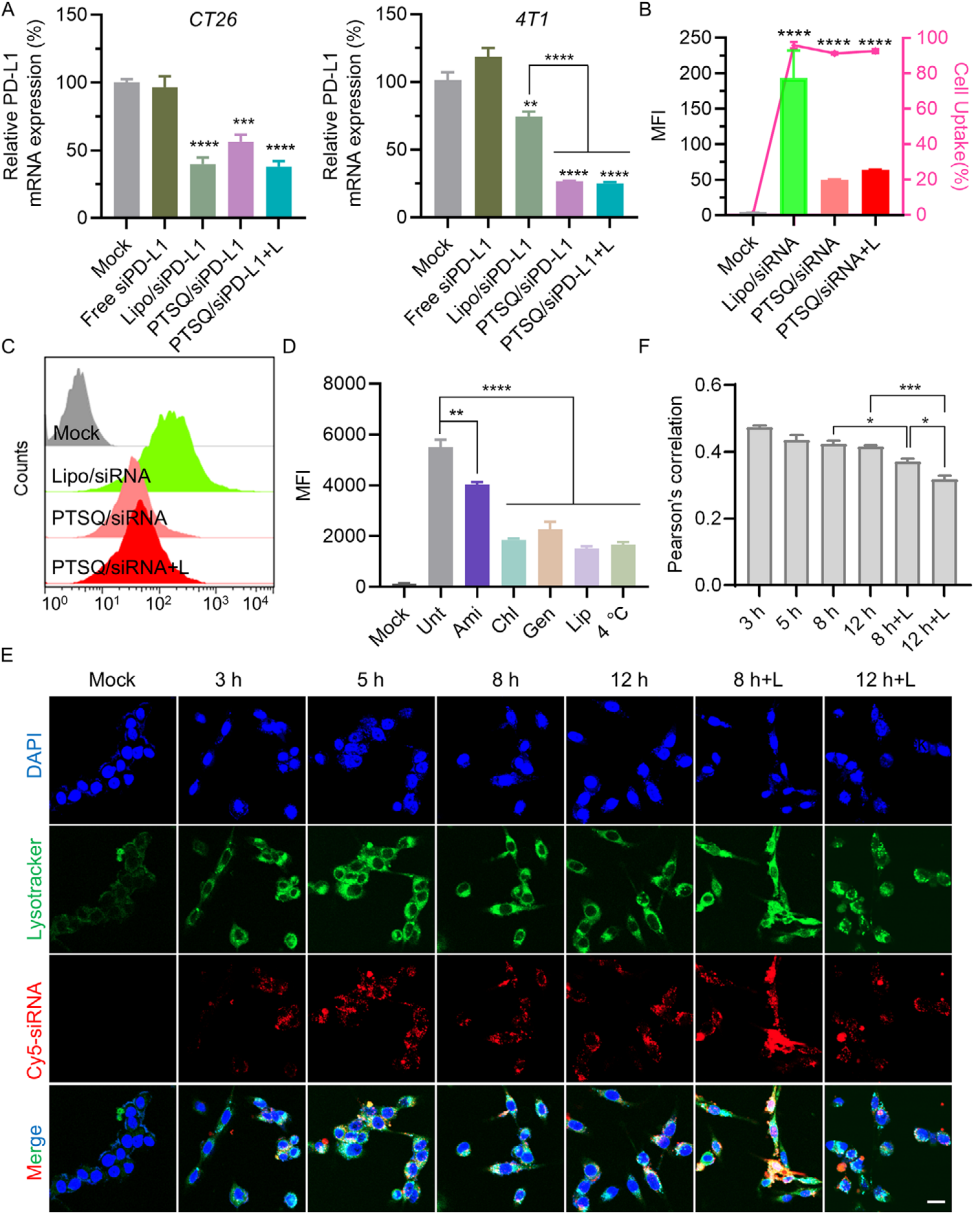
In vitro transfection efficiency and mechanism of PTSQ/siRNA. (A) Evaluation of gene silencing efficiency of PTSQ/siPD‐L1 (w/w = 50:1) at 50 nM siRNA transfection concentration in CT26 and 4T1 cells using qRT‐PCR. (B,C) FACS depicting the cellular uptake of PTSQ/siRNA complex in CT26 cells. (D) FACS showing the inhibition of CT26 cells uptake treated with various inhibitors or at 4°C. The inhibitors included chlorpromazine (simplified by “chl”), amiloride (simplified by “ami”), genistein (simplified by “gen”) and lipid raft (simplified by “lip”). (E) Cellular biodistribution of PTSQ/siRNA complex at various transfection time points, scale bars: 20 µm. (F) Colocalization analysis between Cy5‐siRNA and endosome/lysosome of (E). *p < 0.05, **p < 0.01, ***p < 0.001, ****p < 0.0001.

PD‐L1, a crucial immune checkpoint gene, plays a negative regulatory role in T cell activity across various cancers, including colorectal, breast, and hepatic cancer. To investigate the feasibility of PTSQ/siRNA complexes in cancer immunotherapy, we employed siRNAs targeting the PD‐L1 gene and evaluated the gene silencing efficiency of PTSQ/siPD‐L1 complexes in CT26 and 4T1 cells. As illustrated in Figure 2A and Figure S5, PTSQ/siPD‐L1 effectively suppressed the mRNA and protein expression levels of PD‐L1, regardless of laser irradiation. The gene silencing efficiency mediated by PTSQ delivery was comparable in CT26 cells and superior in 4T1 cells when contrasted with the transfection reagent Lipofectamine 2000 (Lipo).

Next, we assessed the cellular internalization of the PTSQ/siRNA complex using flow cytometry analysis (FACS). Although the mean fluorescence intensity (MFI) of PTSQ/siRNA group was lower than that of Lipo positive control group, the percentage of cell uptake was comparable to the Lipo/siRNA groups, indicating a high transfection efficiency of PTSQ/siRNA (Figure 2B,C). It was noteworthy that the MFI of the PTSQ/siRNA+L group was slightly higher than that of PTSQ/siRNA. Furthermore, the PD‐L1 protein expression in the PTSQ/siRNA+L group was lower than in the PTSQ/siRNA group (Figure S5). Given that the gene silencing efficiency of the PTSQ/siRNA groups was comparable to or superior to that of Lipo/siRNA, we hypothesized that PTSQ/siRNA may possess better lysosomal escape capability than Lipo/siRNA, with laser irradiation enhancing this process. To verify our hypothesis, we first observed the distribution status of PTSQ/siRNA within cells and analyzed the colocalization of siRNA with endosomes/lysosomes. As can be seen from Figure 2E, the PTSQ/siRNA complex was near the lysosomes 3 h after transfection. Initially, it was precisely colocalized with endosomes/lysosomes. However, as the transfection time progressed, siRNA and the lysosome tracer (LysoTracker) gradually separated. The Pearson's correlation coefficient of the PTSQ/siRNA+L (laser irradiation group) was lower than that of the PTSQ/siRNA group, reaching its lowest value at 12 h (Figure 2F). This phenomenon fully illustrates the above situation. These results strongly indicated that laser irradiation could enhance the ability of PTSQ/siRNA to escape from endosomes/lysosomes.

To further elucidate the endocytosis mechanism of the PTSQ/siRNA complex, we employed four distinct inhibitors to block endocytosis pathways: chlorpromazine (blocking clathrin‐mediated endocytosis), amiloride (blocking macropinocytosis), genistein (blocking caveolae‐mediated endocytosis), and lipid raft inhibitor (inhibiting lipid raft‐dependent endocytosis) [31]. Simultaneously, the energy‐dependent cellular uptake was impeded by incubating cells at 4°C. CT26 cells that had been pre‐incubated with the aforementioned endocytosis inhibitors were then treated with the PTSQ/siRNA complex for 3 h. FACS revealed that multiple pathways were implicated in the endocytosis of the PTSQ/siRNA complex, including clathrin‐mediated endocytosis, vesicle‐mediated endocytosis, lipid raft‐dependent endocytosis, energy‐dependent, and micropinocytosis (Figure 2D). Additionally, similar results were obtained through CLSM imaging (Figure S6).

### 2.3 In Vitro ICD Cascade and Cell Apoptosis

**FIGURE 3:**
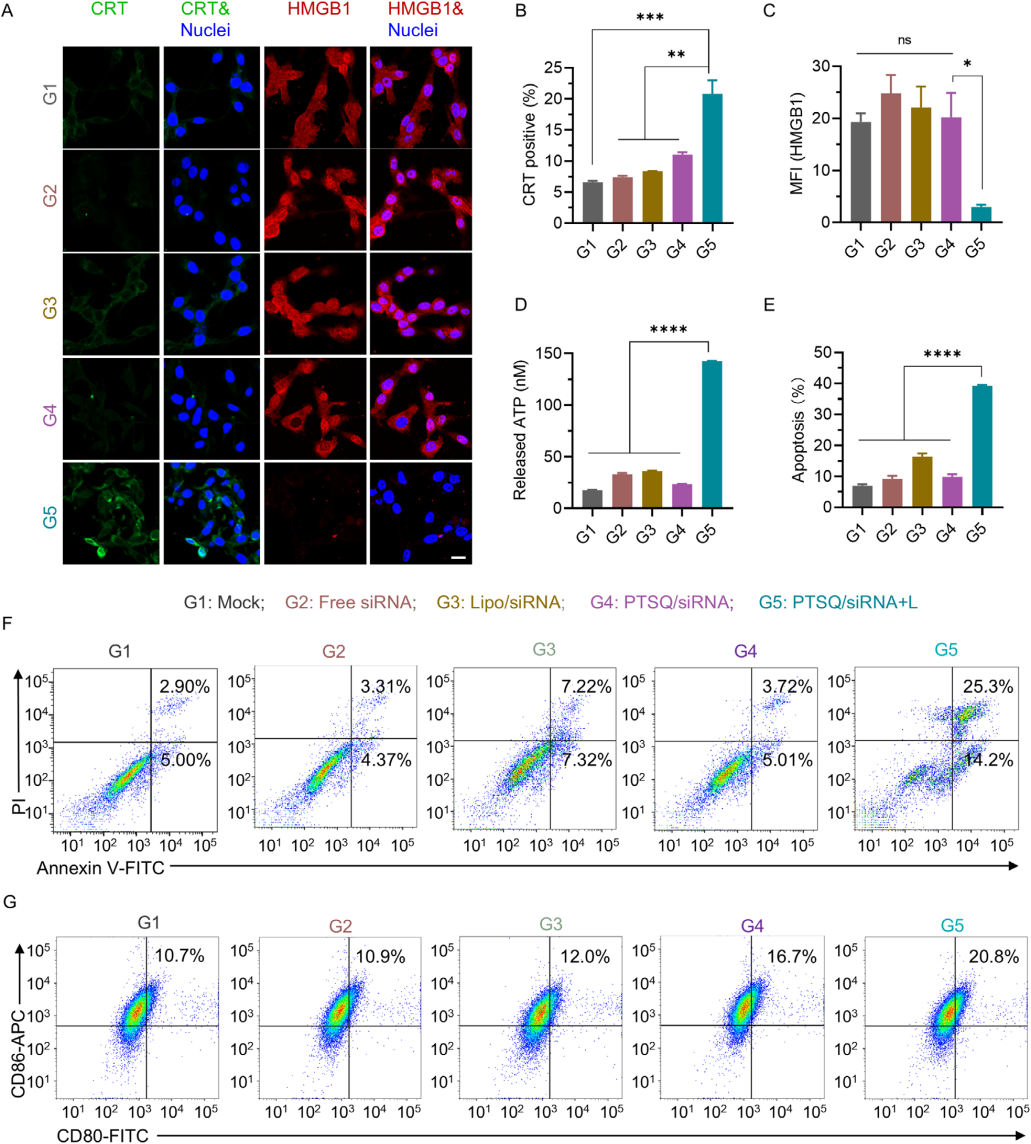
ICD effect induced by PTSQ/siRNA in CT26 cells. (A) CRT exposure and HMGB1 release were visualized using CLSM. Scale bars: 20 µm. (B) CRT exposure analyzed by FACS. (C) Quantification of HMGB1 intensity from CLSM. (D) ATP concentrations were measured using an ATP assay kit. (E,F) Cell apoptosis was assessed by staining cells with Annexin V‐FITC and PI for subsequent FACS. (G) The maturation of DC was evaluated by analyzing the expression of CD80 and CD86 on DC2.4 cells by FACS. *p < 0.05, **p < 0.01, ***p < 0.001, ****p < 0.0001.

The induction of the ICD effect stands as a pivotal bridge between PDT and tumor immunotherapy, heightening anti‐tumor immune responses while impeding tumor growth and metastasis. In order to scrutinize the potential of the PTSQ/siRNA complex in eliciting ICD, we examined key ICD markers, including CRT exposure, HMGB1 release, and ATP secretion. The results from CLSM and FACS revealed that following treatment with the PTSQ/siRNA complex, CRT translocated to the cell surface, with a significantly higher proportion of CRT‐positive cells observed in the laser irradiation group compared to other groups (Figure 3A,B). Measurement of HMGB1 indicated that the PTSQ/siRNA+L group exhibited the lowest retention of HMGB1 within the nucleus, implying the highest release of HMGB1 (Figure 3A,C). Furthermore, extracellular ATP, known to induce T cell differentiation and enhance macrophage and neutrophil chemotaxis (Figure 3D) [11, 32], was significantly elevated in the PTSQ/siRNA+L group, reaching approximately 8.35‐fold compared to others (Figure 3E). In summary, our results underscore the robust capability of the prepared PTSQ/siRNA complex to induce potent ICD effects in in vitro tumor cells.

Next, we investigated whether the ICD effect manifested effective anti‐tumor activity at the cellular level. We employed FACS to assess the extent of cell apoptosis in CT26 cells after various treatments. As shown in Figure 3E,F, the PTSQ/siRNA+L group induced the most severe cell apoptosis, reaching 39.5%. Additionally, we validated this result using the live/dead cell staining method. As depicted in Figure S7, the cells in the PTSQ/siRNA+L group exhibited intense red fluorescence, whereas little red fluorescence was detected in other groups. Moreover, we evaluated the effect of ICD induced by PTSQ/siRNA+L on the maturation of DCs (Figure 3G). The results demonstrated a substantial augmentation in the expression levels of the costimulatory molecules CD80 and CD86 on DC2.4 cells following coculture with CT26 cells treated with PTSQ/siRNA+L. The percentage of mature DCs in the PTSQ/siRNA+L treated group was significantly higher than that in other groups (Figure S8). This finding suggests that immunogenic phototherapy mediated by PTSQ/siRNA+L could effectively promote DC maturation. Overall, laser‐activated PTSQ/siRNA complexes generate ROS, induce an ICD cascade, promote DC maturation, and exhibit a robust in vitro anti‐tumor effect.

### 2.4 In Vivo Distribution and Anti‐Tumor Activity in Colorectal Tumor Model

Stimulated by the favorable anti‐tumor effects in vitro, we shift our focus to investigate the in vivo distribution of PTSQ/siRNA in mice bearing CT26 tumors to assess the retention of the complex in tumor tissues. As illustrated in Figure S9A,B, the fluorescence signals were comparable between the PTSQ/siRNA+L and the PTSQ/siRNA groups at 4 and 6 h post‐intratumoral administration. However, starting from 12 h post‐administration, the fluorescence intensity in the tumors of the PTSQ/siRNA+L group was obviously higher than that of the PTSQ/siRNA group (Figure S9B), with the greatest difference observed at 48 h. Notably, both groups exhibited siRNA accumulation within the tumor for over a week. This suggests that after laser irradiation, more siRNA was released from PTSQ, facilitating endosomal escape and transcytosis of the siRNA. Local administration combined with laser irradiation helps reduce the systemic circulation of PTSQ/siRNA complexes and enhances the therapeutic concentration of the agent in localized tumor tissues.

**FIGURE 4:**
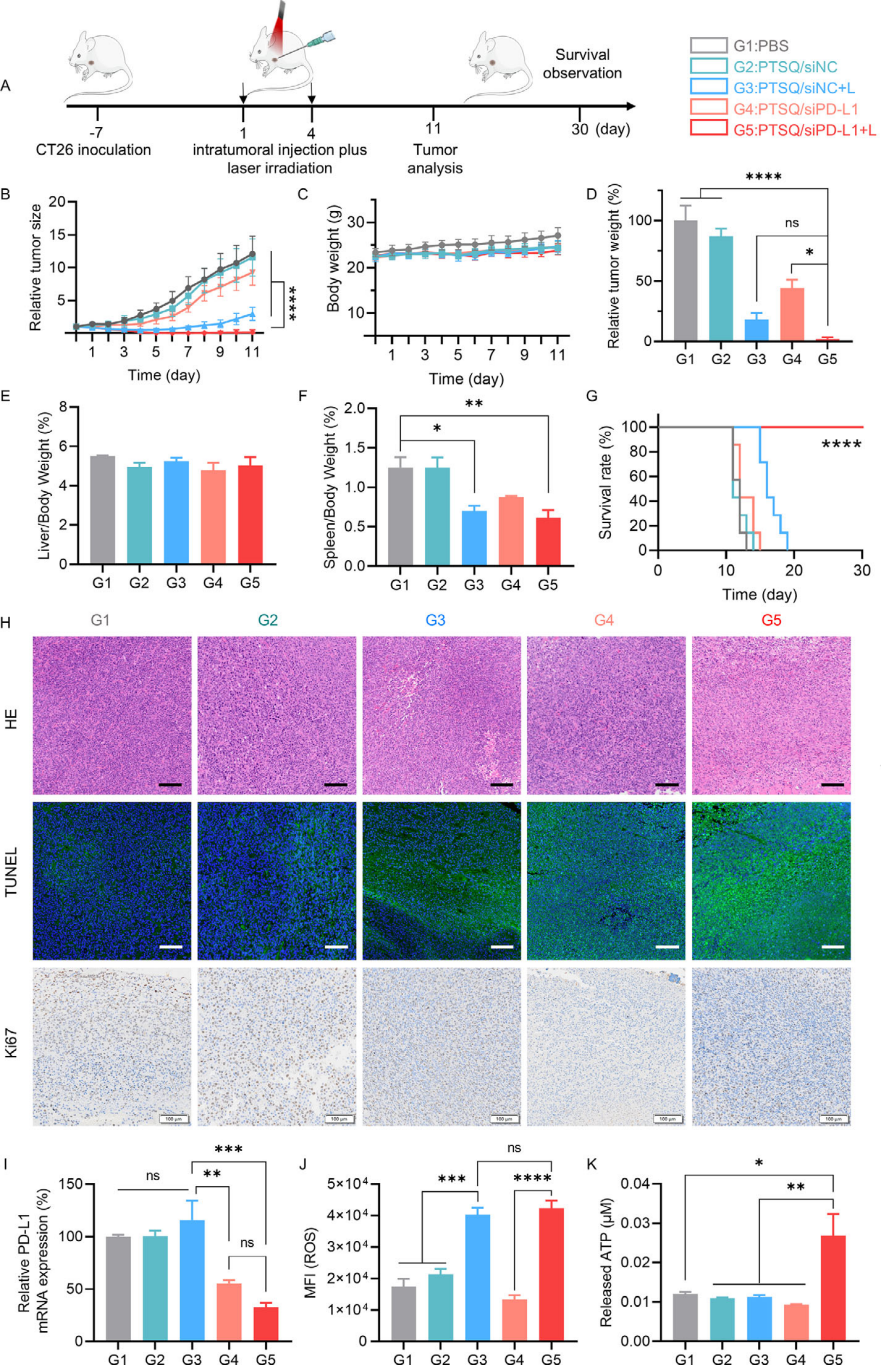
Anti‐tumor efficacy of PTSQ/siRNA complex in CT26 tumor model (*n* = 10). (A) Schematic illustration of tumor treatment and details of treatment groups. Throughout the treatment period, various parameters, including relative tumor size (B), body weight (C), relative tumor weight (D), liver/body coefficient (E), spleen/body coefficient (F), and survival curve (G), were continuously monitored. (H) Representative images of H&E staining, Ki67 staining and terminal deoxynucleotidyl transferase dUTP nick end labeling (TUNEL) in tumor sections. Scale bar: 100 µm. (I) The expression of PD‐L1 mRNA in tumor tissues, determined by qRT‐PCR. (J) ROS generation in tumor tissues was determined using the DHE probe. (K) The amount of ATP released in tumor tissue was determined using the ATP assay kit. Data from (D) to (G) and (I) to (K) were subjected to one‐way ANOVA statistical analysis. *p < 0.05, **p < 0.01, ***p < 0.001, ****p < 0.0001.

Due to the robust induction of ROS and ICD at the cellular level by PTSQ/siRNA complexes, we conducted an in‐depth investigation into their in vivo anti‐tumor efficacy. In Figure 4A, we established a murine colorectal cancer (CT26) model through subcutaneous transplantation of CT26 cells in healthy mice. 1 week later, with tumors reaching 80 mm^3^, mice were randomly assigned to five treatment groups, receiving two intratumoral injections of siRNA at 0.5 mg kg^−1^ dosage. For laser irradiation treatment, tumors underwent a 3‐min exposure to 808 nm laser (power density: 1 W cm^−^^2^) post‐injection.

As depicted in Figure 4B, during the initial treatment phase (day 1 to day 5), none of the treatment groups exhibited notable therapeutic effects. However, from day 6 to day 11, the PTSQ/siPD‐L1+L group displayed a significant inhibition of tumor growth compared to all other groups. Remarkably, the PTSQ/siNC+L group also demonstrated significant inhibition of tumor growth relative to all other groups except PTSQ/siPD‐L1+L (Figure 4D). This phenomenon likely stems from the potent PDT effect induced by PTSQ nanocomplexes. Additionally, the involvement of siPD‐L1 exhibited significant anti‐tumor therapeutic effects. These findings highlighted the pivotal roles of both PTSQ's PDT action and siPD‐L1's ICB effect in murine colorectal cancer immunotherapy.

During the treatment, there were no significant changes in the body weight of mice in any of the treatment groups (Figure 4C). After 11 days of treatment, mice from each group were euthanized, and tumor tissues, along with blood were collected for analysis. As shown in Figure 4E, the liver‐to‐body weight ratio of mice showed little significant change. However, the spleen‐to‐body weight ratio in PBS and PTSQ/siNC groups was slightly higher than in other groups (Figure 4F), suggesting that the other groups were less likely to cause splenomegaly. The survival curve demonstrated that the PTSQ/siPD‐L1+L significantly extended mouse survival in comparison with other treatment groups, showing a similar trend as the results of tumor size and weight (Figure 4G). H&E staining exhibited significant necrosis in the tumor tissues of the PTSQ/siNC+L and PTSQ/siPD‐L1+L groups (Figure 4H). Immunohistochemical Ki67 staining, represented by light brown color, revealed reduced staining in the PTSQ/siNC+L and PTSQ/siPD‐L1+L groups, indicating a notable decrease in tumor cell proliferation compared to the control groups. Consistent findings were observed in TUNEL staining, demonstrating elevated levels of cell apoptosis (green color) and necrosis in the PTSQ/siPD‐L1+L group compared to other treatment groups. In summary, the combination therapy of PTSQ and siPD‐L1 exhibited a robust anti‐tumor effect.

Apart from the anti‐tumor effect, we also evaluated the photodynamic effect, gene silencing efficacy of PD‐L1, and safety profile in different treatment groups of CT26 tumors. As expected, PTSQ/siNC and PTSQ/siNC+L exhibited no inhibitory effect on the expression of PD‐L1 mRNA, whereas the PTSQ/siPD‐L1+L group displayed enhanced gene silencing activity within tumor tissues compared to the PTSQ/siPD‐L1 group (Figure 4I). This enhanced effect should primarily be attributed to the synergistic action of improved tumor accumulation of ROS, and ROS‐induced cytoplasmic release of siPD‐L1. Building on these findings, we further examined the photodynamic efficacy of PTSQ in vivo, with results in Figure 4J indicating that ROS levels were significantly elevated in the PTSQ/siNC+L and PTSQ/siPD‐L1+L groups compared to the other groups. Additionally, histological analyses of major organs revealed that mice with treatment showed no significant histological or pathological changes (Figure S10). Analysis of hematological and serum biochemical indexes in mice after treatment (Figure S11) revealed that all indexes were comparable among all treatment groups and were within or close to normal ranges (the gray shaded region represents the normal range in healthy BALB/c mice). Thus, we considered the treatment with the PTSQ/siRNA complex to be safe under intratumoral injections.

### 2.5 In Vivo ICD Effect and Tumor Microenvironment Immune Priming

**FIGURE 5:**
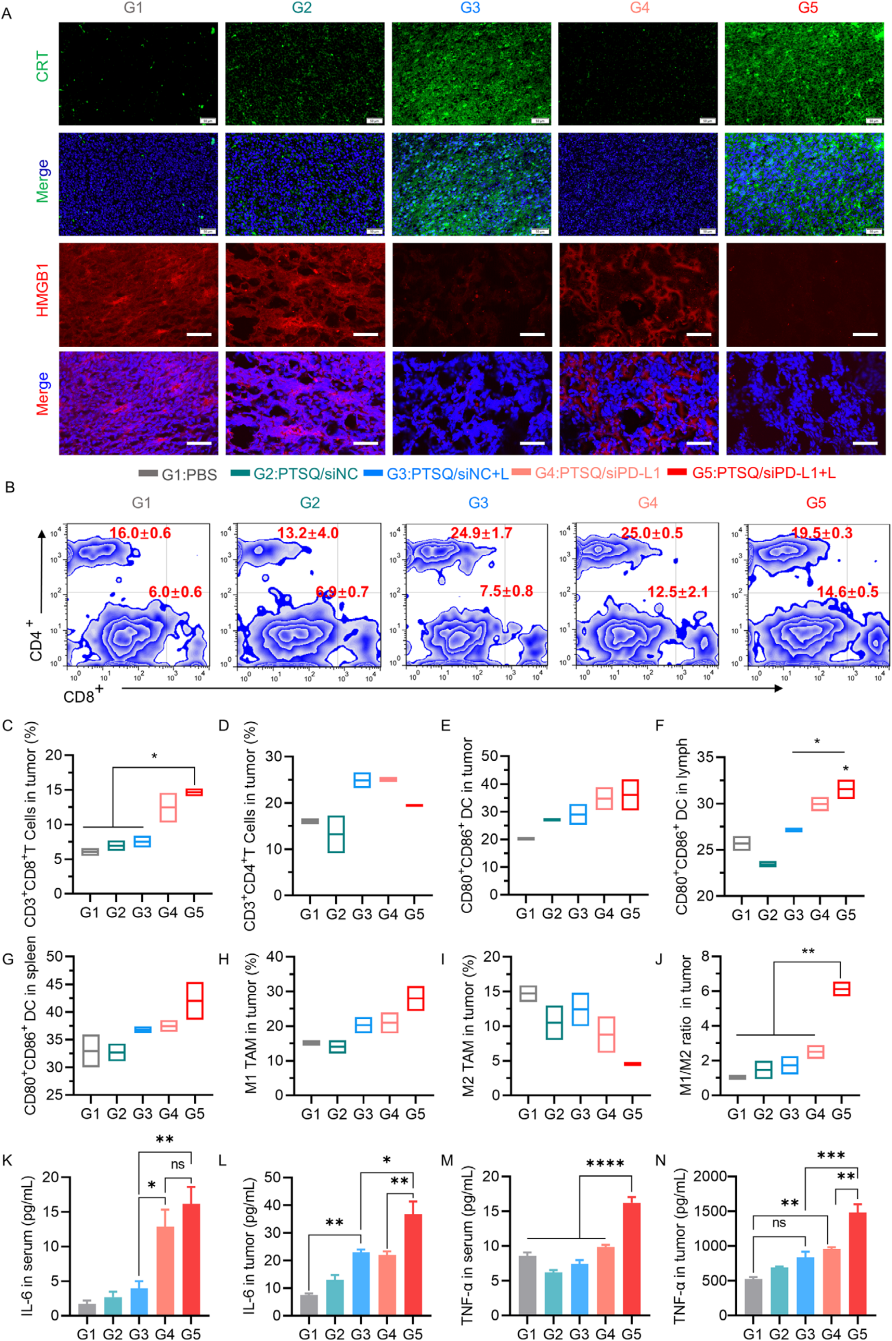
In vivo immune priming analysis of PTSQ/siRNA complex. (A) Immunofluorescence staining for CRT and HMGB1 in tumor tissue sections. Scale bar: 50 µm. (B) FACS of T cells infiltrating the tumors. Quantification of CD8^+^ and CD4^+^ T cell percentages was presented in panels (C, D), respectively. Additionally, the percentages of mature DCs in tumor (E), lymph nodes (F), and spleen (G), as well as the subpopulation of macrophages (H,I) and their ratio (J) in the tumor were quantified and analyzed. (K,L) The expression levels of IL‐6 in serum and tumor, respectively. (M,N) The expression levels of TNF‐α in serum and tumor, respectively. Statistical analysis for data from (C) to (N) was performed using one‐way ANOVA. *p < 0.05, **p < 0.01, ***p < 0.001, ****p < 0.0001.

To investigate whether the PTSQ/siRNA complex could induce ICD effect in vivo, key ICD parameters, including ATP, CRT, and HMGB1, were thoroughly assessed using harvested CT26 tumor tissues. As depicted in Figure 4K, the ATP secretion in the PTSQ/siRNA+L group was significantly elevated, and the CRT signal in this group was also remarkably strong. In stark contrast, the CRT signals in other groups were relatively weak (Figure 5A). Conversely, the red fluorescence signals of HMGB1 within the nuclei of cells treated with the PTSQ/siRNA+L group were notably reduced, providing clear evidence of the occurrence of ICD (Figure 5A). In summary, the PTSQ/siRNA+L complex successfully induced a significant ICD effect in mice by markedly increasing ATP secretion, effectively promoting the release of HMGB1 from the nucleus, and significantly enhancing the exposure of CRT on the cell membrane.

Encouraged by the in vivo ICD effect of the PTSQ/siRNA complex, we investigated the potential for immune priming in the tumor microenvironment. T cell responses within tumors were initially assessed using FACS. As illustrated in Figure 5B–D, PTSQ/siPD‐L1+L exhibited the most significant expansion of CD3^+^CD8^+^ T cells (14.6%), manifesting a significant difference from the PBS, PTSQ/siNC, and PTSQ/siNC+L groups. This indicated that siPD‐L1 also contributed to T cells priming within the tumor, although the population of CD3^+^ CD4^+^ T cells among all groups did not exhibit significant differences.

We then investigated the percentages of mature DCs in the tumors, lymph nodes, and spleens of mice. The results demonstrated an increased percentage of mature DCs in all three tissues under PTSQ/siPD‐L1+L (Figure 5E–G) treatment. Notably, in the spleen, significant differences were observed between the PTSQ/siPD‐L1+L group and the PBS group or PTSQ/siNC+L groups. Specifically, relative to the PBS group and the PTSQ/siNC+L group, the percentage of mature DCs in the PTSQ/siPD‐L1+L group increased by 1.23‐fold and 1.16‐fold, respectively. This observation further supported the crucial role of siPD‐L1 as a stimulator for DC maturation.

Studies have shown that BODIPY‐based photosensitizers produce ROS by the Type I process [33], which occupies an important role in the polarization of macrophages [34]. The aforementioned results for tumor ROS indirectly provided evidence that PTSQ was produced by ROS via a Type I process, as dihydroethidium (DHE) has been identified as a type I probe [35]. Consequently, we measured the percentages of M1‐ and M2‐type macrophages, as well as the M1/M2 macrophage ratios in tumor tissues using FACS. As illustrated in Figure 5H–J, a decrease in the proportion of M2 macrophages (F4/80^+^/CD80^−^/CD206^+^) and an increase in M1 macrophages (F4/80^+^/CD80^+^/CD206^−^) were observed within the tumor tissues. Particularly, the M1/M2 ratio dramatically increased with PTSQ/siPD‐L1+L treatment (Figure 5J). Collectively, these results suggested that adaptive immunity could be induced by PTSQ/siPD‐L1+L in CT26‐tumor‐bearing mice, and this effect was further enhanced through the combination with the siPD‐L1. The combination ultimately resulted in a potent anti‐tumor effect of the PTSQ/siRNA complex.

In tumor immunotherapy, cytokines such as tumor necrosis factor α (TNF‐α) and interleukin‐6 (IL‐6) play crucial roles in immune regulation and inflammatory responses [36]. To comprehensively evaluate the tumor immune effects of PTSQ/siPD‐L1, we next examined the expression levels of TNF‐α and IL‐6 in tumor tissues and serum, respectively. As shown in Figure 5K–N, neither the PTSQ/siNC+L group nor the PTSQ/siPD‐L1 group induced an increase in the expression levels of these cytokines in tumor tissues or serum. However, after treating tumors with the PTSQ/siPD‐L1+L group, the expressions of TNF‐α and IL‐6 were significantly elevated. Notably, compared to the PTSQ/siPD‐L1 group, the PTSQ/siPD‐L1+L group induced a 1.5‐fold increase in TNF‐α expression (Figure 5N) and a 1.7‐fold increase in IL‐6 expression (Figure 5L) in tumor tissues. These results indicate that the PTSQ/siPD‐L1+L group promotes immune activation within the tumor microenvironment, triggering an anti‐tumor immune response.

### 2.6 Tumor Suppression in 4T1‐Xenograft and PDX Tumor Models

**FIGURE 6:**
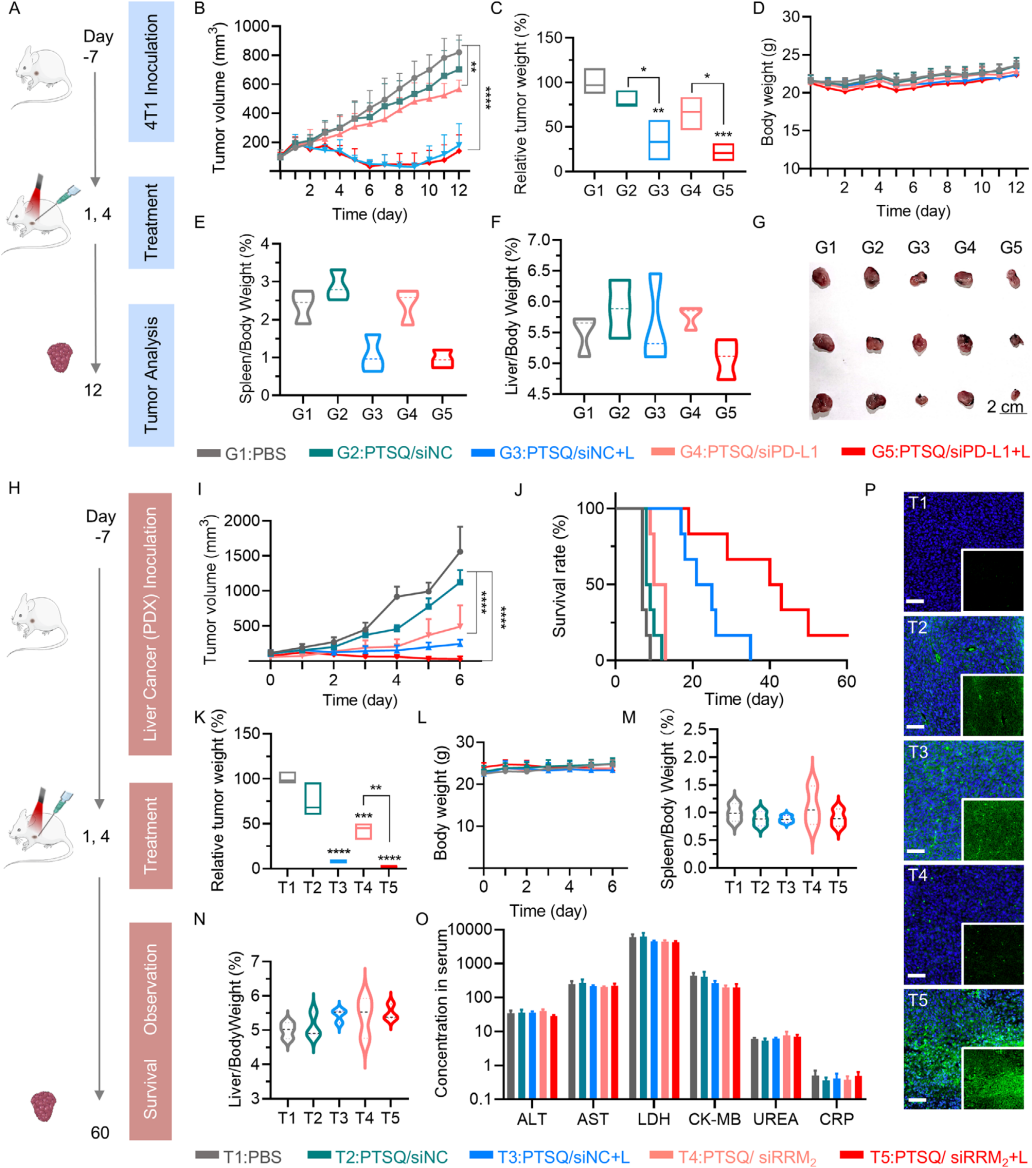
In vivo anti‐tumor efficacy of PTSQ/siRNA complex in 4T1 tumor model and patient‐derived xenograft (PDX) liver cancer model. (A) Schematic illustration of the construction of 4T1 tumor model and the respective treatments. (B) Continuous monitoring of tumor volume. Quantification of relative tumor weights (C), body weight (D), spleen/body weight (E), and liver/body weights (F) for sacrificed mice at the conclusion of the therapy. (G) Image of separated tumors from sacrificed mice and group information. (H) Schematic illustration of the construction of the PDX liver cancer model and the respective treatments. Quantification of tumor volume (I), relative tumor weight (K), body weight (L), spleen/body weight (M), and liver/body weight (N) at the conclusion of therapy. (L) Survival curve of PDX tumor‐bearing mice monitored over 60 days. (O) Analysis of alanine aminotransferase (ALT, U L^−1^), aspartate transaminase (AST, U L^−1^), lactate dehydrogenase (LDH, U L^−1^), creatine kinase isoenzyme‐MB (CK‐MB, U L^−1^), urea nitrogen (UREA, mmol L^−1^), C‐reactive protein (CRP, mg L^−1^) in the serum of mice after treatment on the sixth day. (P) Immunofluorescence staining for CRT in PDX tumor tissues. The larger image was a merged result of TUNEL and DAPI staining, representing an enlarged section from the smaller image. Scale bar: 100 µm. *p < 0.05, **p < 0.01, ***p < 0.001, ****p < 0.0001.

To assess the universality of immunotherapy, we established another tumorigenicity model using 4T1‐Luc cells in mice. As depicted in Figure 6A, BALB/c mice were subcutaneously inoculated with 4T1‐Luc cells a week prior to treatment initiation. Subsequently, mice were randomly assigned to five treatment groups. Notably, PTSQ/siPD‐L1 exhibited a moderate inhibitory effect on tumor growth, whereas PTSQ/siNC+L and PTSQ/siPD‐L1+L demonstrated a significant reduction in tumor volume (Figure 6B,C,G). At the terminal therapeutic time point, the tumor weights index further confirmed this trend, with PTSQ/siNC+L and PTSQ/siPD‐L1+L displaying the smallest tumor weights (Figure 6C). The H&E and TUNEL staining of tumor sections revealed that PTSQ/siNC+L, PTSQ/siPD‐L1, and PTSQ/siPD‐L1+L exhibited tumor cell apoptosis and necrosis to some extent. The CRT exposure also showed the same tendency (Figure S12). No significant differences in body weight, liver/body, spleen/body weight indexes, and levels of serum biochemical parameters were observed across all groups during the treatment period (Figure 6D–F and Figure S13).

Moreover, we established a patient‐derived xenograft (PDX) liver cancer model by transplanting PDX tissue into BALB/C nude mice (Figure 6H). In order to enhance the cancer responsive rate, we introduced siRRM2 in the PTSQ/siRRM2 complex instead of siPD‐L1. The ribonucleotide reductase M2 gene (RRM2) is highly expressed in various tumor cells, leading to abnormal proliferation and malignant transformation of tumor cells [37]. The tumor volume and weight were continuously monitored and recorded over a 6‐day period. Notably, after the second injection on day 4, the therapeutic outcomes exhibited a trend in all groups, with PTSQ/siPD‐L1+L demonstrating the most efficient inhibition of tumor growth.

As the therapy approached its conclusion, the differences among the groups were further magnified, but still aligned with the earlier trend (Figure 6I,K). The survival curve also indicated that the laser irradiation groups displayed the most significant therapeutic benefits, while PTSQ/siRRM2 alone exhibited limited potential for extending survival length (Figure 6J). Since siRRM2 gene silencing was not associated with ICB, we observed enhanced CRT signals and tumor cell apoptosis in the two laser irradiation groups, whereas PTSQ/siRRM2 alone did not show such effects (Figure 6P and Figure S14). This observation indirectly validated that siPD‐L1 could enhance ICD in tumor cells. This effect was achieved by disrupting communication between tumor cells and T cells, thus reinstating T cell‐mediated anti‐tumor activity [38]. Furthermore, we analyzed the body weight, serum biochemical indexes, spleen/body weight, liver/body weight indexes, and H&E staining of mice bearing PDX tumors. These indexes showed no significant differences among the various treatment groups (Figure 6L–O and Figure S14), confirming the safety profile of intratumoral injection with PTSQ nanoparticles and PTSQ/siRNA complexes.

## 3 Discussion and Conclusion

Anti‐PD‐L1 therapy plays a crucial role in tumor immunotherapy, but its responsiveness is influenced by various factors. Some severe malignant tumors, such as pancreatic cancer, glioma, and triple‐negative breast cancer, exhibit relatively low response rates to anti‐PD‐L1 therapy. Even in tumor types responsive to anti‐PD‐L1 treatment, the overall response rate for patients is only about 20%–30% [21]. Numerous studies suggest that modulating the tumor microenvironment, for example, increasing the number of effector T cells in tumor tissue, can enhance the responsiveness and safety of anti‐PD‐L1 therapy [17, 39].

The tumor microenvironment is an extraordinarily complex system, comprising tumor cells, immune cells, stromal cells, and numerous cellular factors. A major challenge in cancer immunotherapy is to modulate the intricate system, shifting the immune suppression state toward immune activation. Additionally, many tumor cells exhibit weak immunogenicity, making them challenging for the immune system to recognize and eliminate, thus limiting the effectiveness of immunotherapy [22, 40]. Enhancing the immunogenicity of tumors to improve recognition and attack by the immune system poses another significant challenge in cancer immunotherapy.

As is widely known, there is a close relationship between PDT and ICD [41]. PDT induces localized cellular damage through the interaction of light at a specific wavelength and a photosensitizer, not only directly destroying tumor cells but also inducing ICD. ICD signaling molecules, such as CRT, HMGB1, and ATP, are released extracellularly after tumor cell death and recognized by immune cells, such as DCs, initiating an anti‐tumor immune response. Thus, by inducing ICD, PDT can activate the body's immune system, increase the number of activated effector cells (such as CD8^+^ T cells, CD4^+^ T cells, and natural killer cells) within the tumor, and generate a stronger anti‐tumor immune effect [11]. However, the maximum absorption wavelength of first and second‐generation photosensitizers used in PDT treatment is generally between 650–800 nm, limiting penetration depth into tumor tissue. In comparison to the previous two generations, photosensitizers that emit light in the NIR II range have lower scattering and absorption in biological tissues, theoretically possessing deeper tissue penetration capabilities and enhancing the anti‐tumor effect. This represents a new direction in the research of PDT and tumor immunotherapy [40, 42].

Building on these observations, the authors proposed a strategy to enhance the immunogenicity of the tumor microenvironment through NIR II‐guided immunogenic phototherapy and PD‐L1 silencing, aiming to achieve robust tumor immunotherapy. A multifunctional polymer, PTSQ, was designed with both absorption and emission capabilities in the NIR region, and promised potential for combination immunotherapy treatment after interaction with siRNA. Specifically, it was confirmed that the PTSQ/siRNA complex could induce ICD effects, which involved exposure of CRT, release of HMGB1 and ATP, thereby eliciting an adaptive immune response to tumor cells. Under laser irradiation, the PTSQ/siPD‐L1 complex exhibited a dual role in suppressing PD‐L1 mRNA expression by facilitating siRNA cellular uptake and lysosomal escape. The efficacy of the combination treatment in suppressing tumor growth was further validated in vivo using CT26 tumor, 4T1‐Luc tumor, and liver cancer PDX models (Figures 4, 5, 6). The data demonstrated the induction of adaptive immunity by the PTSQ/siRNA complex with laser irradiation in three tumor models, further magnified through the use of a combination of immune checkpoint siPD‐L1. This study offers essential insights for designing nucleic acid delivery systems and sets the stage for an outstanding platform in NIR II‐guided cancer immunotherapy. Despite certain limitations in the current research, forthcoming endeavors should prioritize the improvement of tumor penetration capabilities with NIR II‐guided PTSQ and the elucidation of combination therapy mechanisms.

## 4 Materials and Methods

TRIzol Reagent, RNAlater, 1,3‐diphenylisobenzofuran (DPBF), and 3‐[4,5‐dimethylthiazol‐2‐yl]‐2,5‐diphenyltetrazolium bromide (MTT) were procured from Sigma‐Aldrich (St. Louis, MO). Annexin V‐FITC/PI, DCFH‐DA) SYBR Green PCR Mix, and the TUNEL Apoptosis Detection Kit were obtained from Yeasen Biotechnology (Shanghai, China) Co., Ltd. Calcein‐AM/PI, bovine serum albumin, and red blood cell lysis buffer were supplied by Solarbio (Beijing, China). The kit was purchased from Beyotime Biotechnology (Shanghai, China). CD3‐FITC, CD4‐APC, and CD8α‐PE were acquired from Cell Signaling Technology (CST). CD11c‐Percp‐Cy5.5 and CD206‐PE were sourced from Thermo Fisher Scientific. CD80‐FITC, CD86‐PE, and F4/80‐APC were obtained from Biolegend. Anti‐PD‐L1 antibody, anti‐GAPDH antibody, calreticulin polyclonal antibody, CoraLite488‐conjugated Goat Anti‐Rabbit IgG(H+L), HMGB1 monoclonal antibody, and CoraLite594–conjugated Goat Anti‐Mouse IgG(H+L) were purchased from Proteintech. All the siRNAs used in this study, including siPD‐L1, siNC, and Cy5‐labeled siRNA, were provided by Suzhou Ribo Life Science Co., Ltd. (Jiangsu, China) (Table S1). Additionally, all primers were procured from BioSune Co., Ltd. (Shanghai, China).

### 4.1 Preparation of PTSQ Polymer

The synthesis of PTSQ was conducted in two steps. In the first step, precisely weighed 20.00 mg of M1, 20.72 mg of 2,2'‐(propane‐2,2‐diylbis(sulfanediyl)) bis(ethan‐1‐ol) (DSB), 47.81 mg of L‐lysine diisocyanate, and 22.15 mg of 2,2‐bis(bromomethyl)‐1,3‐propanediol were added to 5 mL anhydrous *N*,*N*‐dimethylformamide, and the reaction proceeded at 50°C for 12 h. Subsequently, 110.67 mg of m‐PEG_5000_‐OH was added, and the reaction continued for an additional 12 h. Then, the reaction mixture was dialyzed against water using a dialysis bag with a cutoff of 7000 MW. The resulting product was then freeze‐dried to obtain polymer P1.

In the second step, 60 mg of P1 polymer and 20 mg of M5 were weighed and added to 5 mL anhydrous *N, N*‐dimethylformamide, and the reaction took place at 50°C for 12 h. The reaction mixture was once again dialyzed against water using a dialysis bag with a cutoff of 7000 MW, followed by freeze‐drying to obtain the PTSQ polymer.

### 4.2 Preparation of PTSQ Nanomicelles and PTSQ/siRNA Complex

PTSQ nanomicelles were prepared using the traditional nanoprecipitation method. Specifically, 10 mg of PTSQ polymer material was dissolved in a 0.5 mL solution of DMF. This solution was then added dropwise to 2 mL of water, with continuous stirring at 1000 rpm using a magnetic stirrer for 30 min. Subsequently, the solution was dialyzed against water using a dialysis bag with a cutoff of 7000 MW for 24 h to remove excess DMF, resulting in the formation of PTSQ micelles.

The PTSQ micelles were then mixed with siRNA through electrostatic adsorption. To fix the siRNA content, different masses of PTSQ micelles were mixed with siRNA solution in a 1:1 volume ratio at room temperature, and the mixture was allowed to stand for 30 min to obtain the PTSQ/siRNA complexes.

### 4.3 Agarose Gel Retardation Assay

The siRNA loading capacity of the PTSQ nanomicelles was assessed through agarose gel electrophoresis experiments. Initially, a 2% agarose gel was prepared, and Gelred staining solution was added to the gel. Subsequently, different PTSQ/siRNA complex samples with varying mass ratios were loaded onto the agarose gel and subjected to electrophoresis for 20 min, with free siRNA serving as the control group. The electrophoresis was conducted at a voltage of 120 V. Following electrophoresis, a gel imaging system was employed for ultraviolet imaging and photographs were taken for analysis.

### 4.4 In Vitro Release of siRNA

To investigate the kinetic behavior of siRNA release from PTSQ/siRNA complexes in vitro, we measured the amount of siRNA released from the PTSQ/siRNA complex under laser irradiation for 4 h. In this experiment, Cy5‐labeled siRNA was used to form complexes with PTSQ. At room temperature, the PTSQ/Cy5‐siRNA was stirred continuously at 500 rpm, while the PTSQ/Cy5‐siRNA+L group was irradiated by 808 nm laser with 2 W cm^−2^ intensity when stirring. At predetermined time points, 100 µL of the solution was sampled and dialyzed against PBS for over 2 h using a dialysis tube with a molecular weight cut‐off of 100 KD. After dialysis, the Cy5 fluorescence intensity was measured using a microplate reader at an excitation wavelength of 640 nm and an emission wavelength of 680 nm. The amount of RNA released at each time point was then calculated. The formula for calculating the RNA release amount (M1) at each time point is *M*_1_ = *M*_2 _/ *M*_0_ × 100%, where *M*_2_ represents the Cy5 fluorescence intensity measured at each time point, and *M*_0_ represents the Cy5 fluorescence intensity in the initial solution. The cumulative RNA release amount at a specific time point is the sum of the release amounts at that point and all preceding time points.

To accurately reflect the release of siRNA from PTSQ/Cy5‐siRNA complex in the tumor cell microenvironment, we added 50 mM of hydrogen peroxide to the PBS buffer to simulate the tumor cell environment and then carried out another set of studies on the release of siRNA. The studies were also conducted under two conditions, that is, with laser irradiation and without it. The specific implementation methods were the same as those mentioned above.

### 4.5 Stability Evaluation

The PTSQ/siRNA complexes were stored at room temperature and 4°C for 14 days, respectively. At specified time points, a certain amount of complex solution was taken out and measured for particle size using a DLS instrument (Zetasizer 3000HS, Malvern, UK).

### 4.6 MTT Assay

Briefly, CT26 cells were seeded in each well of a 96‐well plate and incubated for 24 h at 37°C. Subsequently, the cells were treated with free siRNA, Lipo/siRNA, and PTSQ/siRNA complexes at different mass ratios. After 3 h incubation, the PTSQ/siRNA+L groups were exposed to 808 nm laser light for 4 min at a power density of 2 W cm^−2^. After 24 h incubation, the growth medium was removed, and MTT reagent (100 µg mL^−1^) was added for 4 h incubation at 37°C. The resulting mitochondrial MTT crystals were dissolved in DMSO (100 µL) for 10 min at 37°C. Finally, the absorbance at 540 and 650 nm was measured using a microplate reader.

### 4.7 ROS Detection

The generation of extracellular ROS was assessed using a 1,3‐diphenylisobenzofuran (DPBF) probe, characterized by a reduction in its UV–vis absorption at 420 nm upon reaction with ROS. A 10 µL solution of PTSQ micelles (3 mg mL^−1^) was added into 190 µL DPBF solutions (132.5 mg L^−1^). The UV–vis absorption spectrum was immediately measured between 300 and 600 nm using a microplate reader, and this value was recorded as the 0‐second data. Subsequently, another set of samples was exposed to 808 nm laser light at a power density of 2 W cm^−2^, and the UV–vis absorption spectra of each sample were measured under different irradiation durations (0–300 s).

Furthermore, the generation of cellular ROS was evaluated using the DCFH‐DA probe. CT26 cells were individually treated with free siNC, Lipo/siNC, and PTSQ/siNC complexes for 3 h at 37°C in Opti‐MEM. Then, the DCFH‐DA was added into medium and cultured for 30 min. The laser exposure CT26 cell group was irradiated with an 808 nm laser for 4 min (2 W cm^−2^) and subsequently observed and imaged using fluorescence microscopy.

### 4.8 Live and Dead Cell Staining and Cell Apoptosis Detection

First, CT26 cells were seeded in a 6‐well plate at a density of 2 × 10^5^ cells per well and cultured overnight. The medium was then replaced with Opti‐MEM, and free siNC, Lipo/siNC, and PTSQ/siNC complexes were individually added to the medium and transfected for 3 h. The group with laser irradiation was exposed to laser light (2 W cm^−2^) for 4 min. Subsequently, the medium was replaced with complete culture medium, and cells were further cultured for an additional 24 h. For the live/dead cell staining assay, cells were stained with Calcein AM/PI for 30 min, and observations and recordings were made using CLSM. For the cell apoptosis experiment, transfected CT26 cells were collected by centrifugation, and Annexin V‐FITC/PI double staining was performed for 10 min for FACS analysis.

### 4.9 Intracellular Distribution and Lysosomal Escape

To analyze lysosomal escape, CT26 cells were transfected for 3, 5, 8, and 12 h, respectively. For laser irradiation groups, the cell was exposed to laser light (2 W cm^−2^) for 4 min after 3 h post transfection. Following transfection, the cells were stained for 30 min with LysoTracker Green and then fixed using a 4% formaldehyde solution for 10 min at room temperature. Subsequently, the fixed cell was stained DAPI for 30 min and then examined using CLSM. Colorization analysis between Cy5 and LysoTracker Green was conducted using the ImageJ software.

### 4.10 Endocytosis Mechanism

CT26 cells were preincubated with various endocytosis inhibitors (chlorpromazine: Blocking clathrin‐mediated endocytosis, amiloride: Inhibiting macropinocytosis, genistein: Blocking caveolae‐mediated endocytosis, and lipid raft methyl‐*β*‐cyclodextrin (m‐*β*‐cd): Inhibiting lipid raft‐dependent endocytosis) for 30 min prior to treatment with the PTSQ/siRNA complex. Simultaneously, to inhibit the energy‐dependent endocytosis of the PTSQ/siRNA complex, CT26 cells were put at 4°C. Following a 3 h transfection, cells underwent staining with LysoTracker Green for 30 min, followed by Hoechst 33342 staining for 10 min, and were subsequently visualized using LSM. Simultaneously, an additional parallel set of experiments was carried out, and the MFI was quantified through FACS.

### 4.11 In Vitro Gene Silencing Potency

Real‐time PCR was employed to assess the efficiency of gene silencing by the PTSQ/siPD‐L1 complex, gauging the relative levels of PD‐L1 mRNA. Briefly, cells were plated at a density of 1 × 10^5^ mL^−1^ in six‐well plates and cultured overnight in complete medium. Subsequent to transfection and 24 h incubation, total RNA extraction was carried out using the Trizol reagent, followed by cDNA synthesis and PCR amplification. PD‐L1 mRNA levels were then normalized to endogenous GAPDH mRNA levels, with the specific primers used detailed in Table S1 of the Supporting Information.

To evaluate the impact of siRNA‐mediated silencing on PD‐L1 protein expression, western blotting was performed. Post‐transfection and cell lysis with RIPA lysis buffer (Beyotime, Shanghai, China), total protein content was quantified using a BCA kit (Lot^#^CW0014, CWBIO, Beijing, China). Equivalent protein amounts were separated on a 10% SDS polyacrylamide gel and transferred to a nitrocellulose membrane. After 1 h of blocking with 5% bovine serum albumin (BSA) at RT, the membranes were incubated overnight at 4°C with primary antibodies targeting PD‐L1 or GAPDH. After multiple washes with PBST (PBS buffer with 0.1% Tween 20 addition), the membranes underwent 1 h incubation with the horseradish peroxidase (HRP)‐conjugated secondary antibody. Finally, chemiluminescence reagents (ECL) were applied, and images were captured using a multi‐imaging system (Tanon, Shanghai, China).

### 4.12 In Vitro ICD Efficacy

Immunostaining was employed to assess CRT exposure and HMGB1 release. Initially, CT26 cells post‐transfection were fixed using a 4% formaldehyde solution for 10 min at room temperature. Following this, fixed cells were blocked with 5% goat serum for 1 h at RT, and incubated with anti‐CRT or anti‐HMGB1 antibodies in 5% goat serum overnight at 4°C. After three PBS washes, cells were incubated with the designated secondary antibody for 1 h in the dark. In the subsequent step, nuclei were stained with DAPI for 5 min and washed three times before observation with CLSM. For ATP secretion detection, cell culture supernatants were collected, and extracellular ATP contents were assessed using an ATP assay kit.

### 4.13 Measurement of DC Maturation

The effect of PTSQ/siRNA on DC maturation was measured in vitro by using the transwell system. Specifically, immature DC2.4 cells were cultured in the lower compartments. The upper compartments contained CT26 cells subjected to different treatments. The PTSQ/siNC+L group was exposed to laser light (2 W cm^−2^) for 4 min following the transfection procedure. Then, DC2.4 cells were incubated with the residues of CT26 cells for 48 h. After staining with anti‐CD80‐FITC and anti‐CD86‐APC antibodies, DC2.4 cells were analyzed by FACS.

### 4.14 Anti‐Tumor Efficacy in CT26 Tumor Model

All animal procedures were approved by the Institutional Animal Care and Use Committee (IACUC) of Beijing Institute of Technology and performed in accordance with the guidelines and policies (approval number BIT‐EC‐SCXK (Beijing) 2019‐0010‐M‐2021010).

To investigate the therapeutic effects of PTSQ/siRNA complexes in CT26 tumors, we first established a subcutaneous tumor model in mice. One week before the start of the experiment (day‐7), 1 × 10^6^ CT26 cells were injected subcutaneously into the right dorsal side of female BALB/c mice (6–8 weeks). When the tumor volume reached approximately 80 mm^3^ (day 0), the mice were randomly divided into five groups (*n* = 10). On days 1 and 4, five formulations were intratumorally injected at a dose of 0.5 mg kg^−1^ siPD‐L1, including PBS (G1), PTSQ/siNC (G2), PTSQ/siNC+L (G3), PTSQ/siPD‐L1 (G4), and PTSQ/siPD‐L1+L (G5). The laser‐treatment groups underwent irradiation with 808 nm laser (1.0 W cm^−2^, 3 min) 6 h post‐injection. Tumor size, body weight, and animal survival were recorded daily. The tumor volume was calculated using the formula: volume = length × width^2^ / 2. Animals were considered “dead” when the tumor size exceeded 2000 mm^3^. On study day 5, part of the tumor tissue was collected to test for ROS according to the Tissue Reactive Oxygen Species (ROS) test kit (BBoxiProbe DHE ROS, BB‐470515). At the conclusion of the study, we collected blood or serum samples for routine blood tests and biochemical parameter examinations. Tumor tissues, as well as major organs including the spleen, kidney, liver, lung, and heart, were harvested. We recorded the weights of the tumor, liver, and spleen, and organ coefficients were calculated based on their respective weights to evaluate the effects of different treatments on tissue changes. Tumor tissues and major organs were fixed in a formaldehyde solution for the preparation of paraffin sections, H&E staining, CRT, HMGB1, Ki67, and TUNEL immunohistochemistry staining.

### 4.15 Tumor‐Infiltrating Immune Cell Analysis and Cytokine Profiling

Six days post‐CT26 tumor treatment, the tumor‐bearing mice were sacrificed, and tumors, spleens, and tumor‐draining lymph nodes were harvested and minced to obtain single‐cell suspensions. The cells were then collected by centrifugation at 300 ×*g* for 5 min. After lysing the cells with RBC lysis buffer for 15 min, they were collected again by centrifugation at 300 × *g* for 5 min to remove any residual RBC lysis buffer. Single‐cell suspensions were subjected to FACS using antibodies against surface markers for CD4^+^ T or CD8^+^ T cells (CD3, CD4, CD8α), DCs (CD11c, CD80, CD86), and M1 or M2 macrophages (F4/80, CD80, CD206).

The level of cytokines, including TNF‐α and IL‐6, in the tissue and serum was determined by ELISA assay. Briefly, tumor tissues and blood were retrieved at 6 days post‐treatment. The tumor tissues were homogenized at 4°C for 2 min and centrifuged at 4°C, 1000 g for 10 min, and the blood was also centrifuged at 4°C, 1000 g for 10 min after spontaneous clotting at RT for 1 h. Both supernatants of tumor tissues and blood were then collected for ELISA assay (Beijing Solarbio Science & Technology Co., Ltd.), according to the manufacturer's instructions.

### 4.16 Anti‐Tumor Efficacy in 4T1 Tumor Model

The process of establishing the 4T1 tumor‐bearing mouse model is highly similar to that of the CT26 tumor model, with *n* = 9. In this experimental setup, the treatment groups, dosages, and procedures align with the CT26 anti‐tumor experiment. Tumor volume was continually measured during the treatment period and at the endpoint (day 12), mouse weights, as well as the weights of major organs such as the tumor, liver, and spleen, were recorded. Blood samples were collected for biochemical parameter analysis. H&E staining was performed on tumor tissues and major organs to examine for pathological changes. Immunohistochemical staining for CRT and TUNEL was conducted on tumor tissues to assess immune cell death and apoptosis in the tumor cells.

### 4.17 Anti‐Tumor Efficacy in PDX Tumor Model

For the PDX (patient‐derived xenograft) tumor model, one week before the start of the treatment, PDX tumor blocks were transplanted into the right dorsal subcutaneous region of healthy female BALB/c‐nu mice aged 6–8 weeks. When the subcutaneous PDX tumors reached approximately 100 mm^3^, mice were randomly divided into five groups (*n* = 9). The treatment groups included: PBS (T1), PTSQ/siNC (T2), PTSQ/siNC+L (T3), PTSQ/siRRM2 (T4), and PTSQ/siRRM2+L (T5). Dosages and treatment procedures were consistent with the aforementioned two tumor models. On the sixth day of treatment, three mice were randomly selected from each treatment group, sacrificed, and relevant tissues and blood were collected for analysis. Tumors and major organs were weighed, subjected to H&E staining, and analyzed with CRT and TUNEL staining.

### 4.18 Data Analysis

All data are presented as mean ± SD in this study. Statistical analysis was conducted by using GraphPad Prism 9.5 software. One‐way or two‐way ANOVA tests were used to determine whether there was a significant difference at *p* < 0.05.

## Author Contributions

Conceptualization: Yuanyu Huang, Haihua Xiao, and Yuquan Zhang. Methodology: Yuquan Zhang, Jie Wang, Tian Zhang, and Dongsheng Tang. Investigation: Yuquan Zhang, Jie Wang, Tian Zhang, Dongsheng Tang, Haiyin Yang, Shuai Guo, and Yuchuan Fan. Formal analysis: Yuquan Zhang, Jie Wang, Tian Zhang, Caixia Sun, Haihua Xiao, Yuanyu Huang, and Yuhua Weng. Data curation: Yuquan Zhang, Jie Wang, Tian Zhang, Yuanyu Huang, and Yuhua Weng. Visualization: Yuquan Zhang, Jie Wang, Yuanyu Huang, and Yuhua Weng. Writing – original draft: Yuquan Zhang, Jie Wang, and Yuhua Weng; Writing – review and editing: Yuanyu Huang and Yuhua Weng. Funding acquisition: Yuanyu Huang and Yuhua Weng. Supervision: Yuanyu Huang and Yuhua Weng.

## Conflicts of Interest

Yuanyu Huang is the founder of Rigerna Therapeutics. Yuanyu Huang is a member of the *Exploration* editorial board, and he was not involved in the handling or peer review process of this manuscript. The other authors declare no conflicts of interest.

## Supporting information




**Supporting file 1**: exp270055‐sup‐0001‐SuppMat.docx.

## Data Availability

All data are available in the main text or the supplementary materials.
