# Supplementary material for: NIR II‐Guided Photoactivatable Silencing Polyplex Boosts Cancer Immunotherapy
Source: Exploration (Beijing). 2025 May 30;5(5):20240047. doi: 10.1002/EXP.20240047 (PMC12561200; doi:10.1002/EXP.20240047)
Supplement: Supplementary file 1 — Supporting file 1: exp270055‐sup‐0001‐SuppMat.docx. [file EXP2-5-20240047-s001.docx]

# Supporting Information

**NIR II-guided photoactivatable silencing polyplex boosts cancer immunotherapy**

*Yuquan Zhang ^a,#^, Jie Wang ^a,#^, Tian Zhang ^a^, Dongsheng Tang ^b,c^, Haiyin Yang ^a^, Shuai Guo ^a^, Yuchuan Fan ^a^, Caixia Sun ^d^, Haihua Xiao ^b,c^, Yuanyu Huang ^a,e,f,^*, Yuhua Weng ^a,e,f,^**

^a^ School of Life Science; School of Interdisciplinary Science; Aerospace Center Hospital; Key Laboratory of Molecular Medicine and Biotherapy; Key Laboratory of Medical Molecule Science and Pharmaceutics Engineering; Beijing Institute of Technology, Beijing 100081, China.

^b^ Beijing National Laboratory for Molecular Sciences, Laboratory of Polymer Physics and Chemistry, Institute of Chemistry Chinese Academy of Sciences, Beijing 100190, China.

^c^ University of Chinese Academy of Sciences, Beijing 100049, China.

^d^ School of Chemistry, Chemical Engineering & Biotechnology, Nanyang Technological University, 637371, Singapore.

^e^ School of Medical Engineering; School of Interdisciplinary Science; Affiliated Zhuhai People's Hospital; Beijing Institute of Technology (BIT), Zhuhai 519088, China.

^f^ Advanced Technology Research Institute, Beijing Institute of Technology (BIT), Jinan 250101, China.

.

*^#^* Y. Z. and J. W. contributed equally to this work.

^*^ Corresponding author: [yyhuang@bit.edu.cn](mailto:yyhuang@bit.edu.cn) (Y.H.); [yhweng@bit.edu.cn](mailto:yhweng@bit.edu.cn) (Y.W.)

Table S1. siRNA sequences.

| Name (siRNA) | Sequence (5′-3′) | Target gene |
| --- | --- | --- |
| siNC | (s) CCUUGAGGCAUACUUCAAAdTdT | none |
|  | (as) UUUGAAGUAUGCCUCAAGGdTdT |  |
| siPD-L1 | (s) AGACGUAAGCAGUGUUGAA | PD-L1 |
|  | (as) UUCAACACUGCUUACGUCUCC |  |


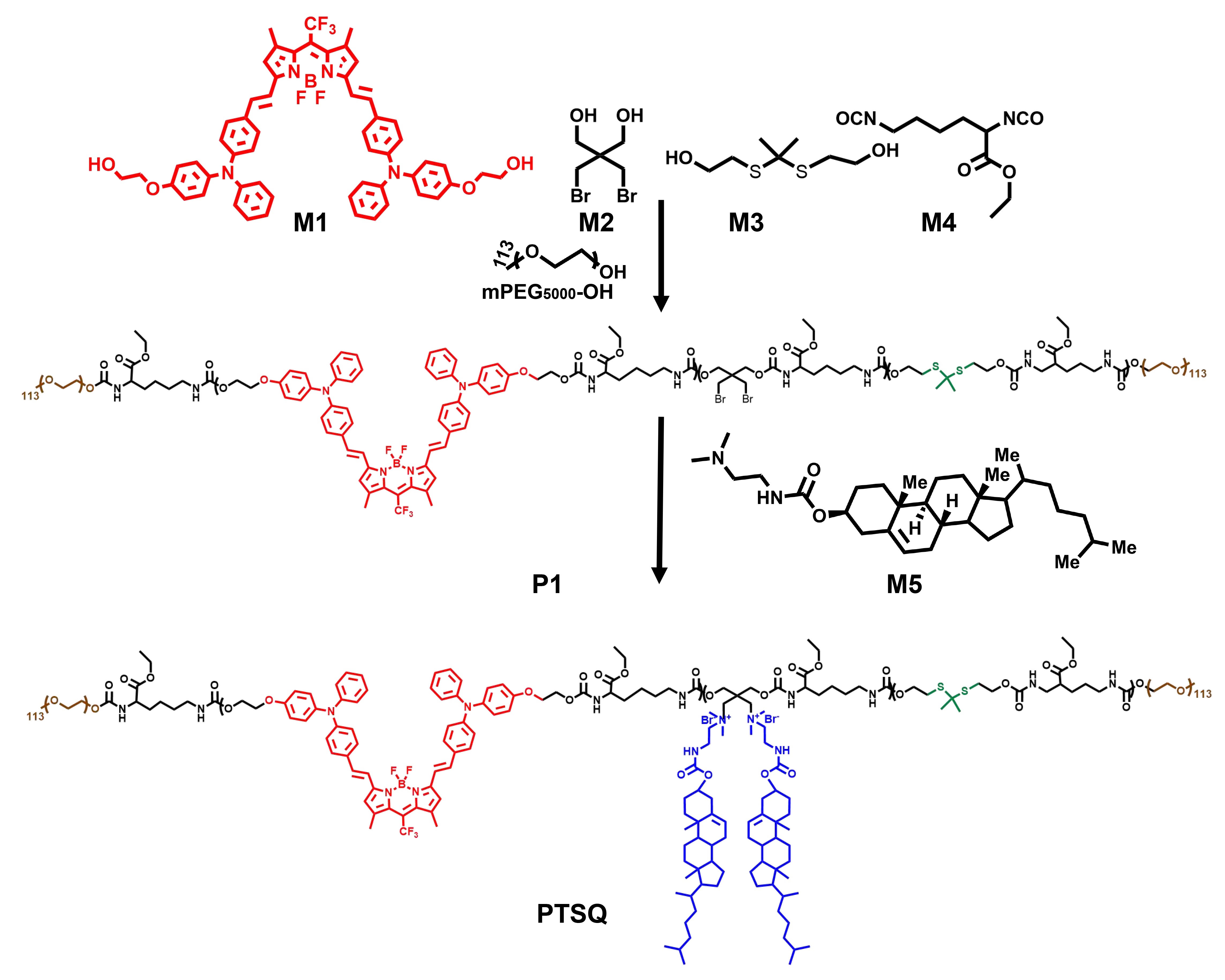


Figure S1. The synthesis route of PTSQ polymer.


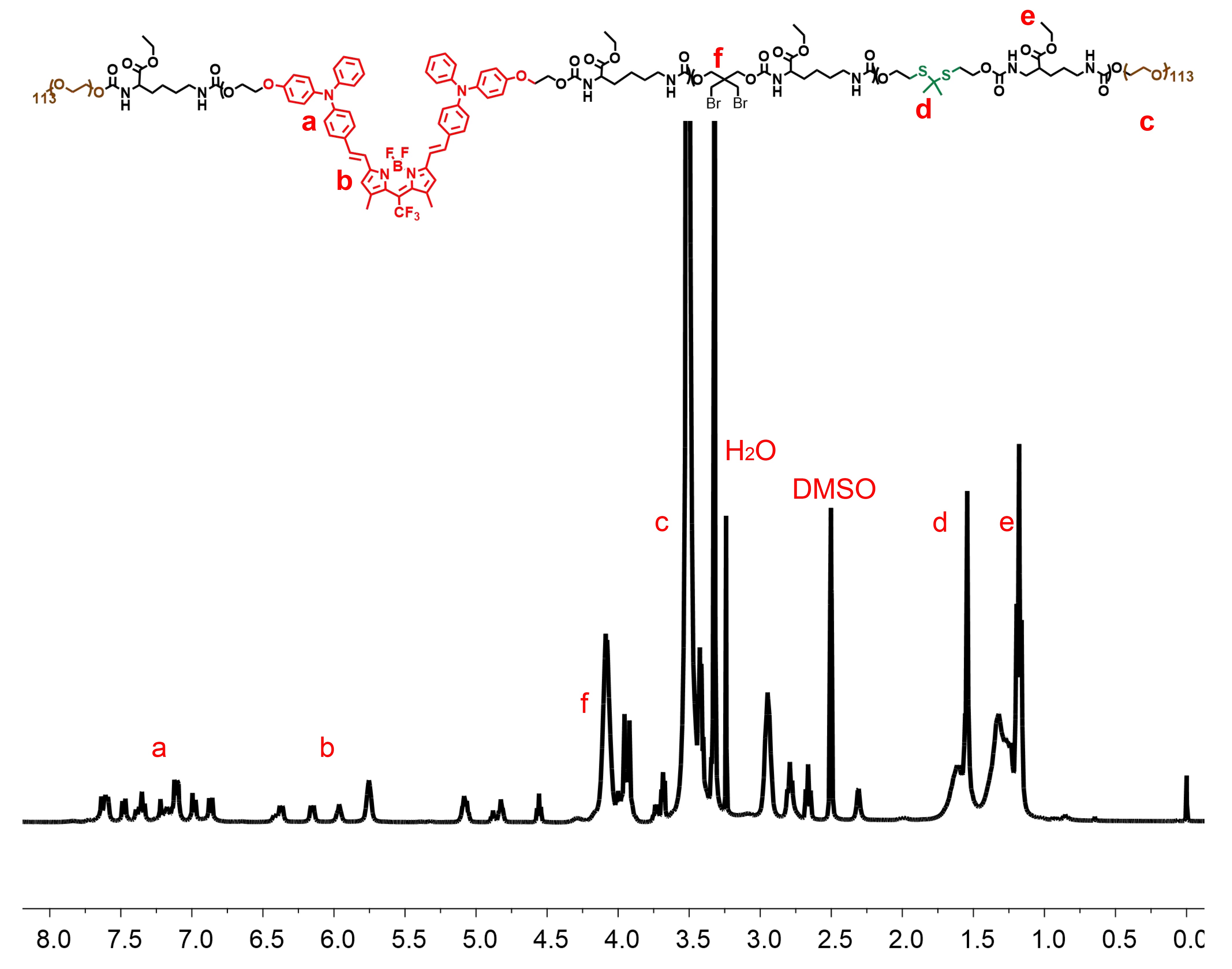


Figure S2. ^1^H NMR spectrum of P1 polymer.


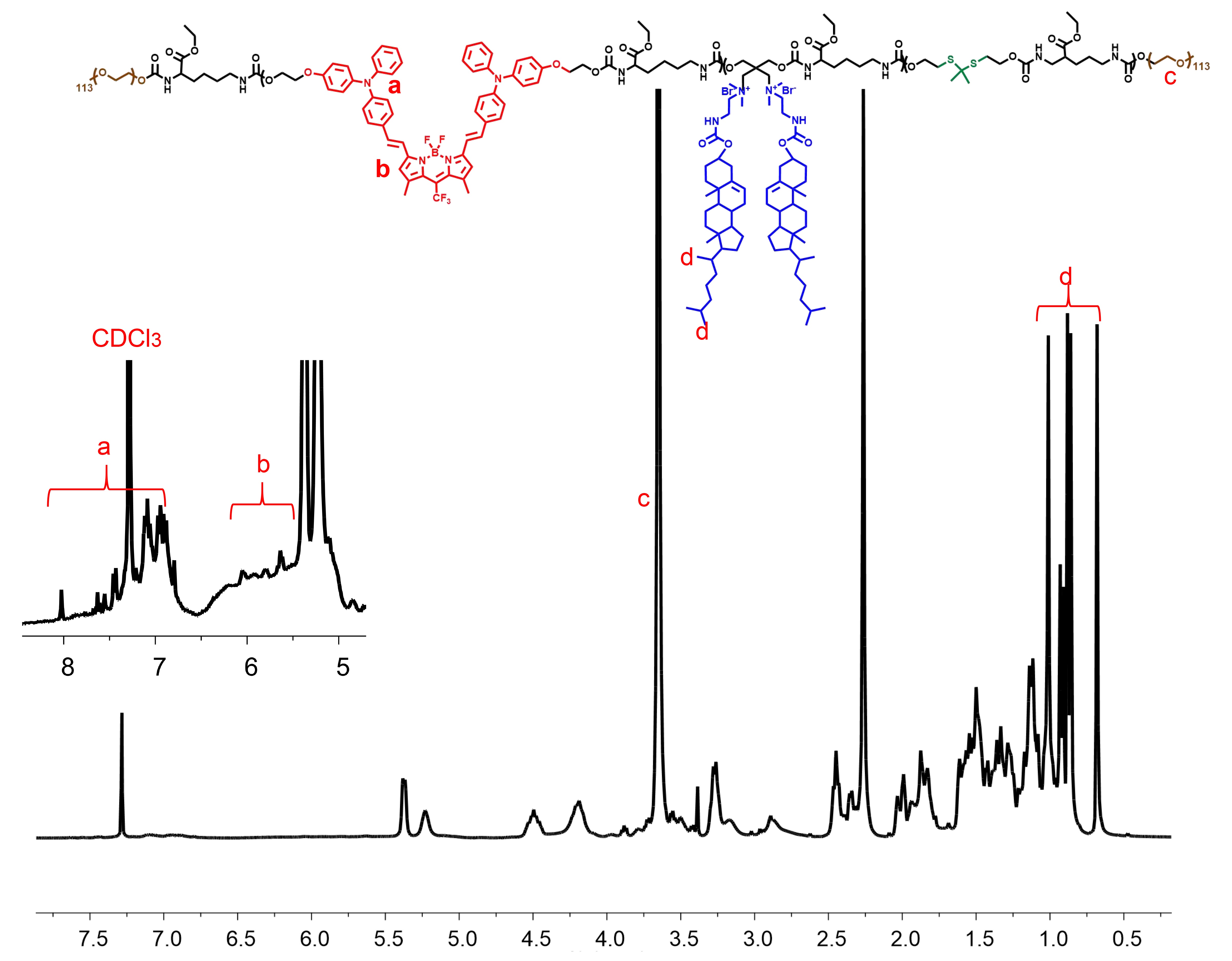


Figure S3. ^1^H NMR spectrum of PTSQ polymer.


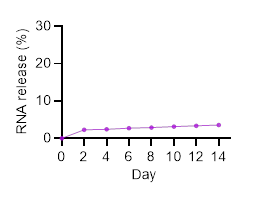


Figure S4. *In vitro* release of siRNA from PTSQ/siRNA during storage for two weeks at RT.


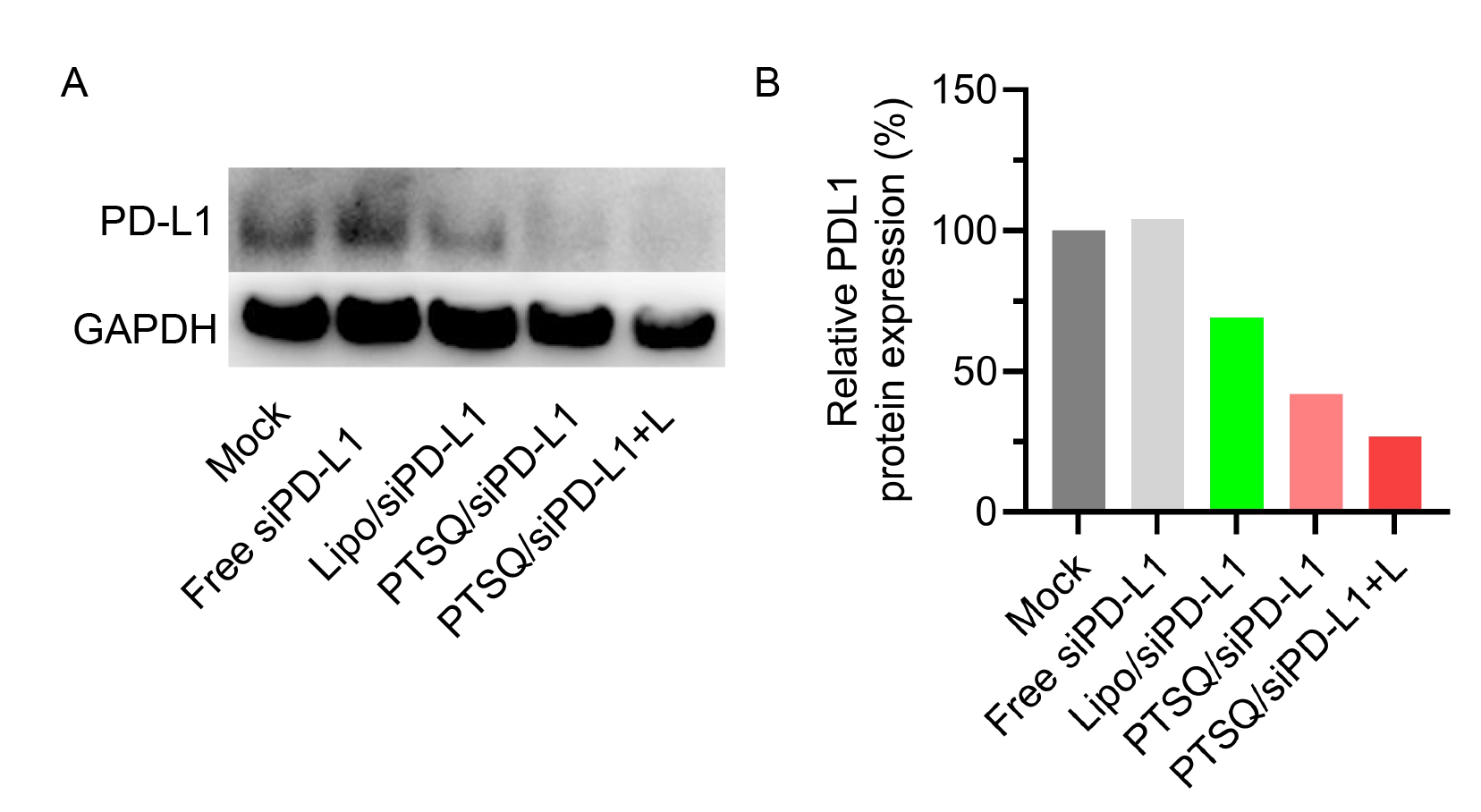


Figure S5. Western blotting analysis of PD-L1 protein expression in CT26 cells post transfection for 24 h. “(L)” represented laser irradiation.


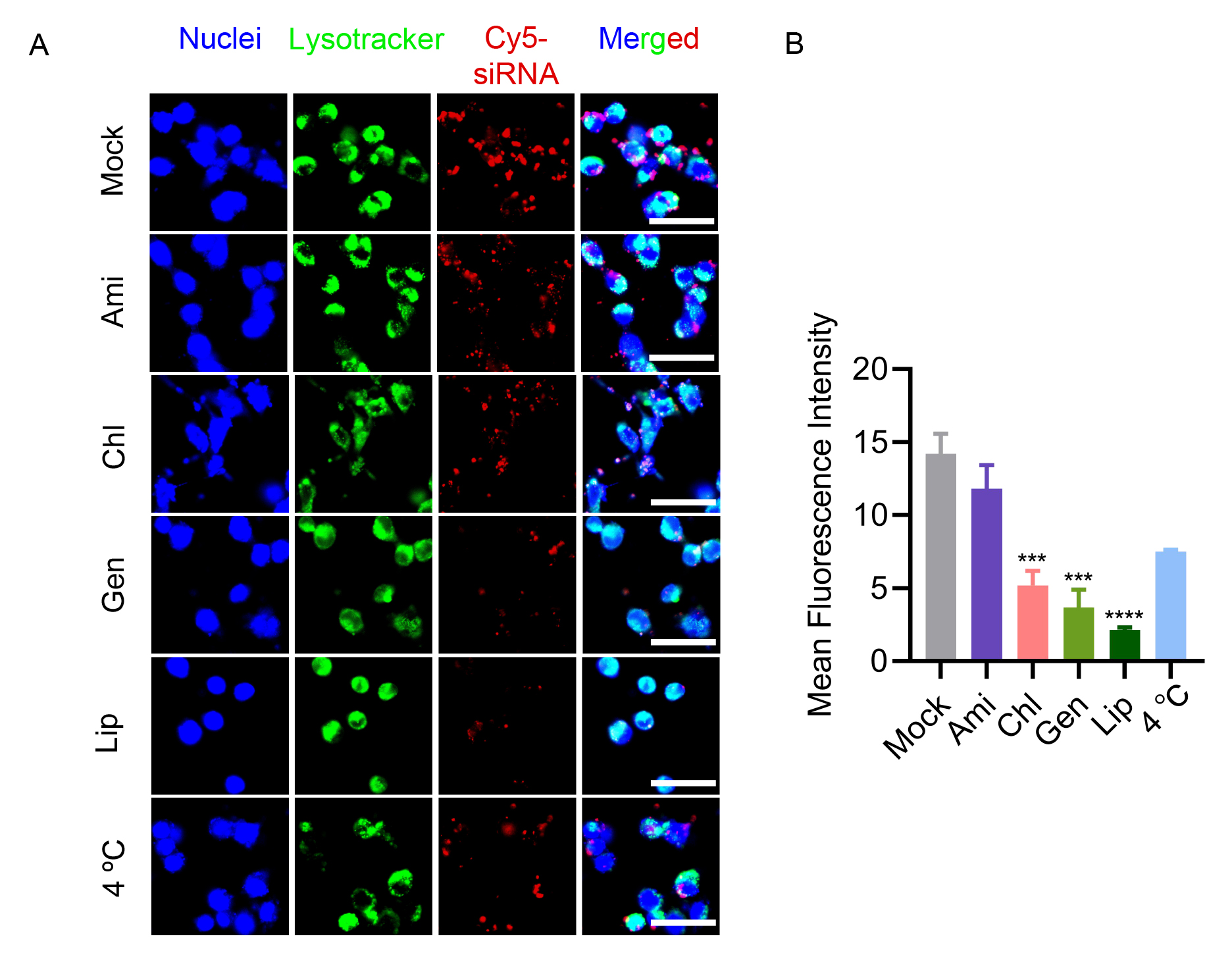


Figure S6. Endocytosis mechanism analysis of PTSQ/siRNA complex. (A) CLSM images of CT26 cell being treated with different endocytosis inhibitors. Scale bar: 50 μm. (B) Quantitative mean fluorescence intensity (MFI) of Cy5 signal by using FACS. **p < 0.01, ***p < 0.0005, ****p < 0.0001.


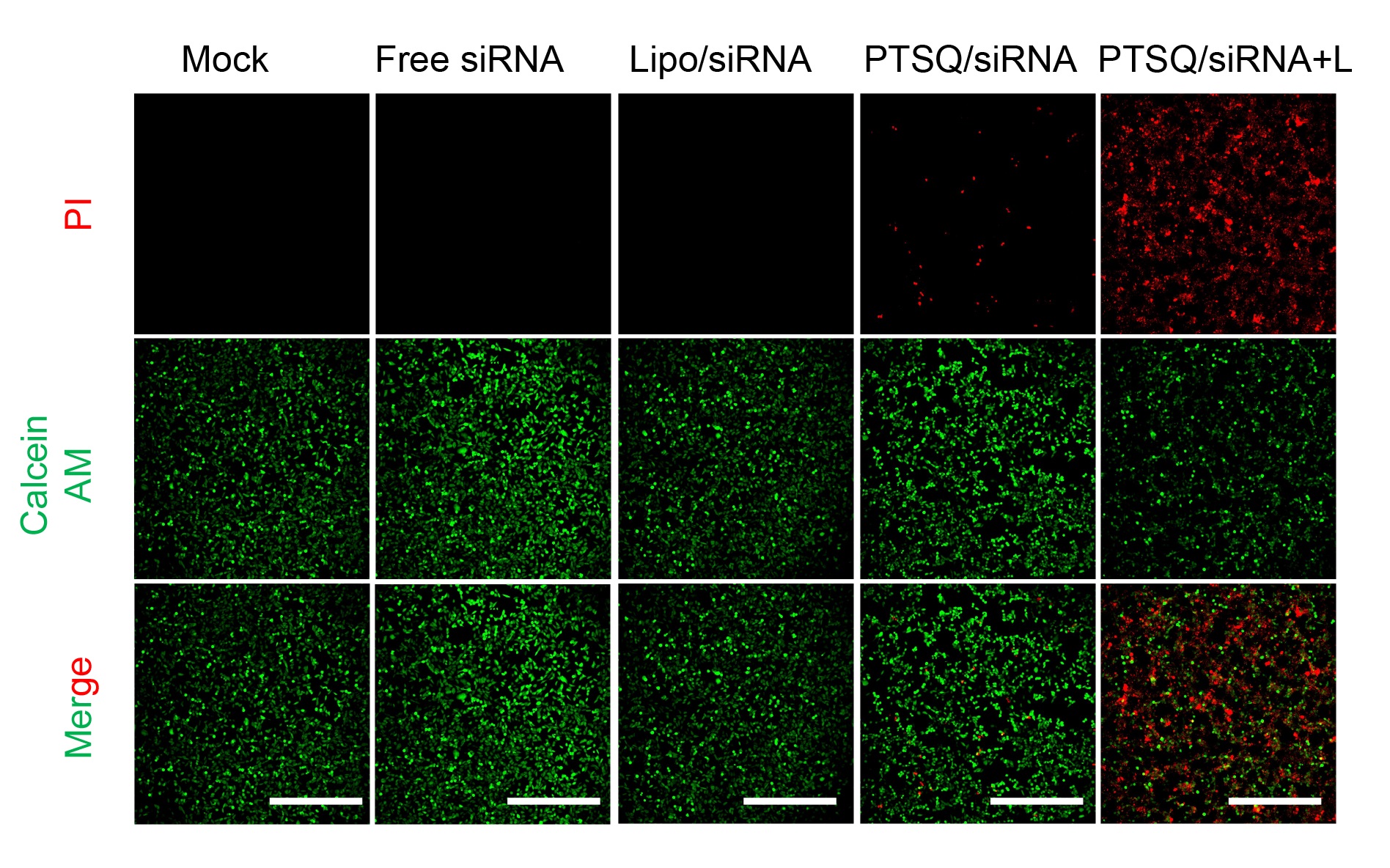


Figure S7. Calcein-AM and PI staining of CT26 cells after being treated with various groups. Scale bar: 250 μm.


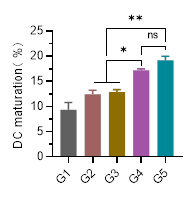


Figure S8 *In vitro* DC maturation quantification.


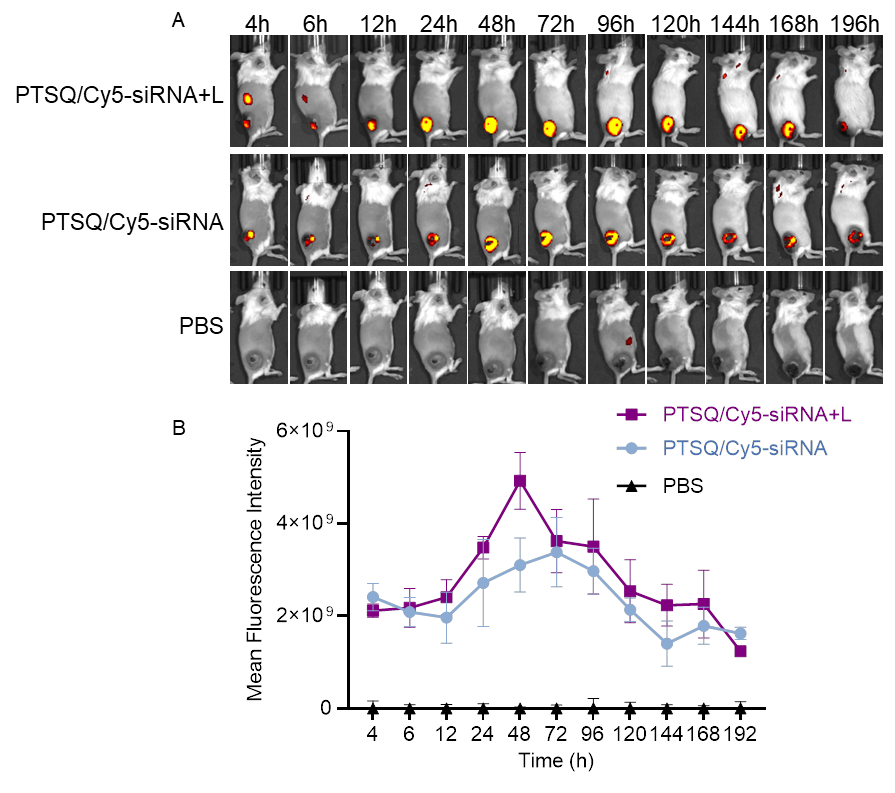


Figure S9. (A) Time-dependent biodistribution of PTSQ/Cy5-siRNA complex in CT26-tumors. (B) Quantitative analysis of tumor fluorescence intensity. The laser-treatment groups underwent irradiation with 808 nm laser (1.0 W cm^-2^, 3 minutes) 6 hours post-injection.


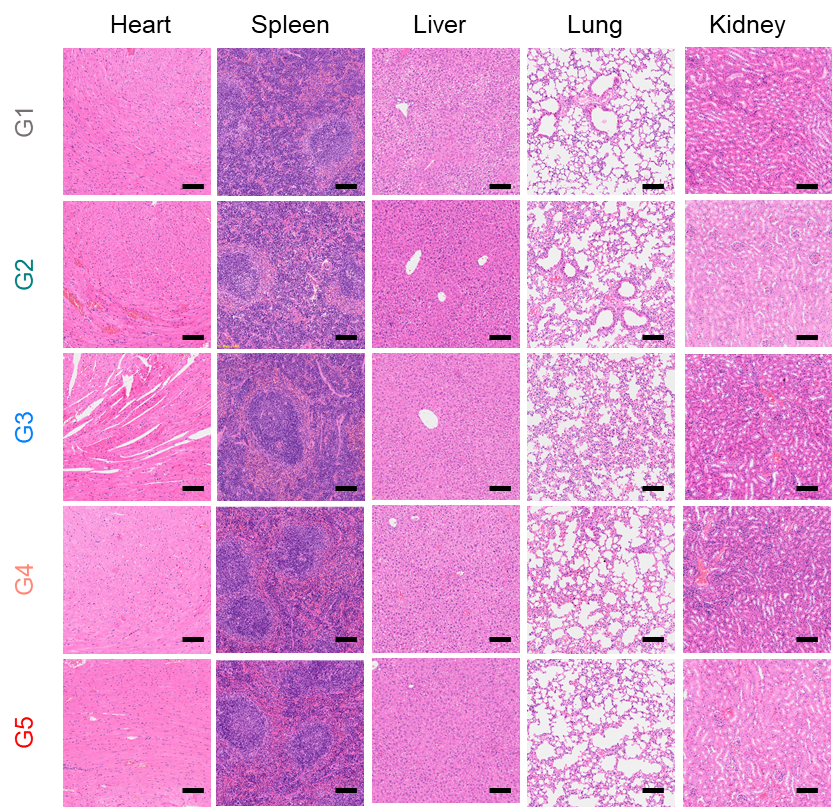


Figure S10. H&E staining of major organs from sacrificed CT26 tumor-bearing mice under treatment. Scale bar: 100 μm.


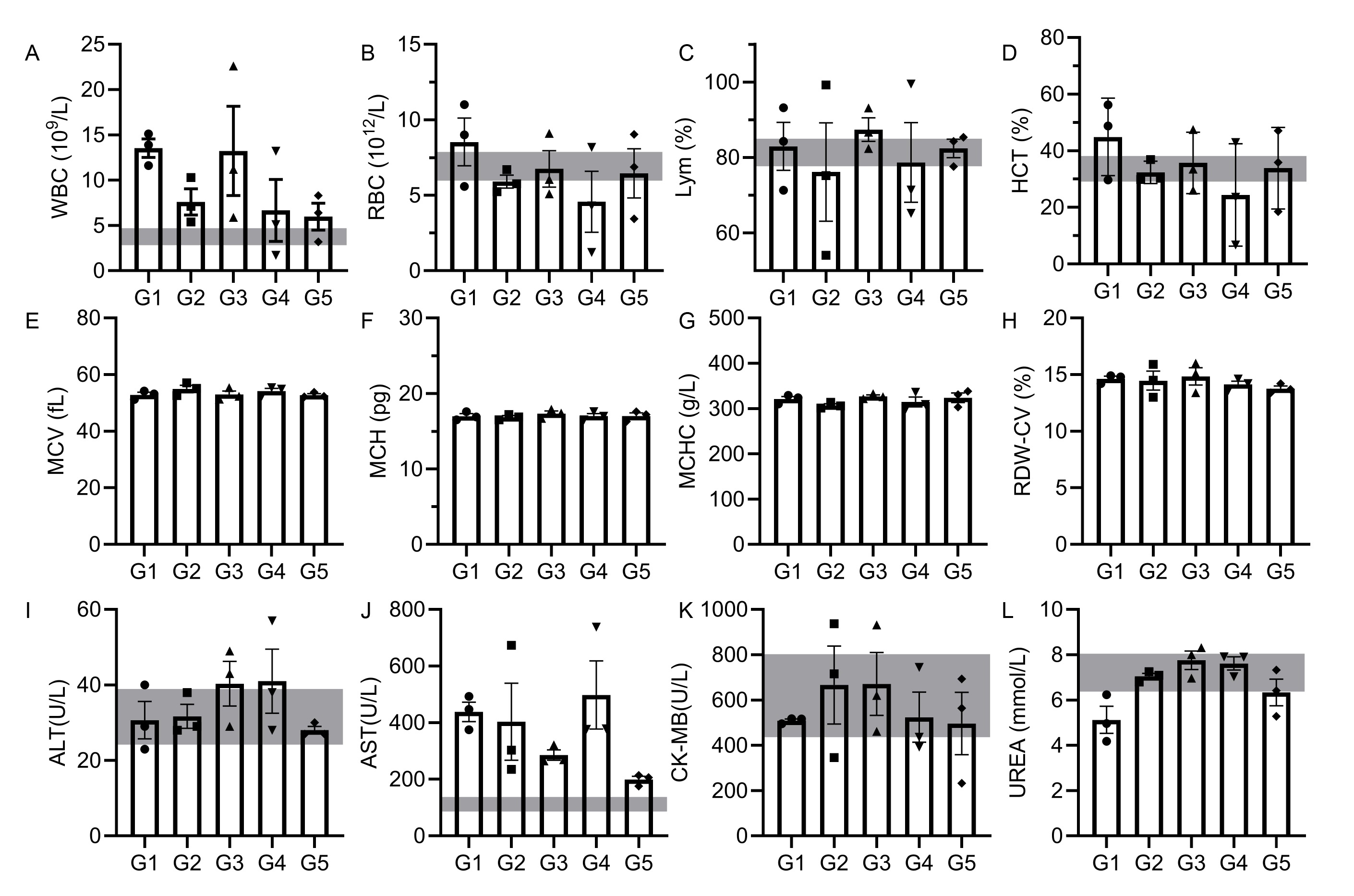


Figure S11. Physiological and biochemical levels of CT26 tumor-bearing mice after treatment. (A-H) Hemocyte analysis in mice. (I-L) Serum biochemical parameters. G1: PBS, G2: PTSQ/siNC, G3: PTSQ/siNC+L, G4: PTSQ/siPD-L1, G5: PTSQ/siPD-L1+L. The gray shaded region represents the normal range in healthy BABL/c mice.


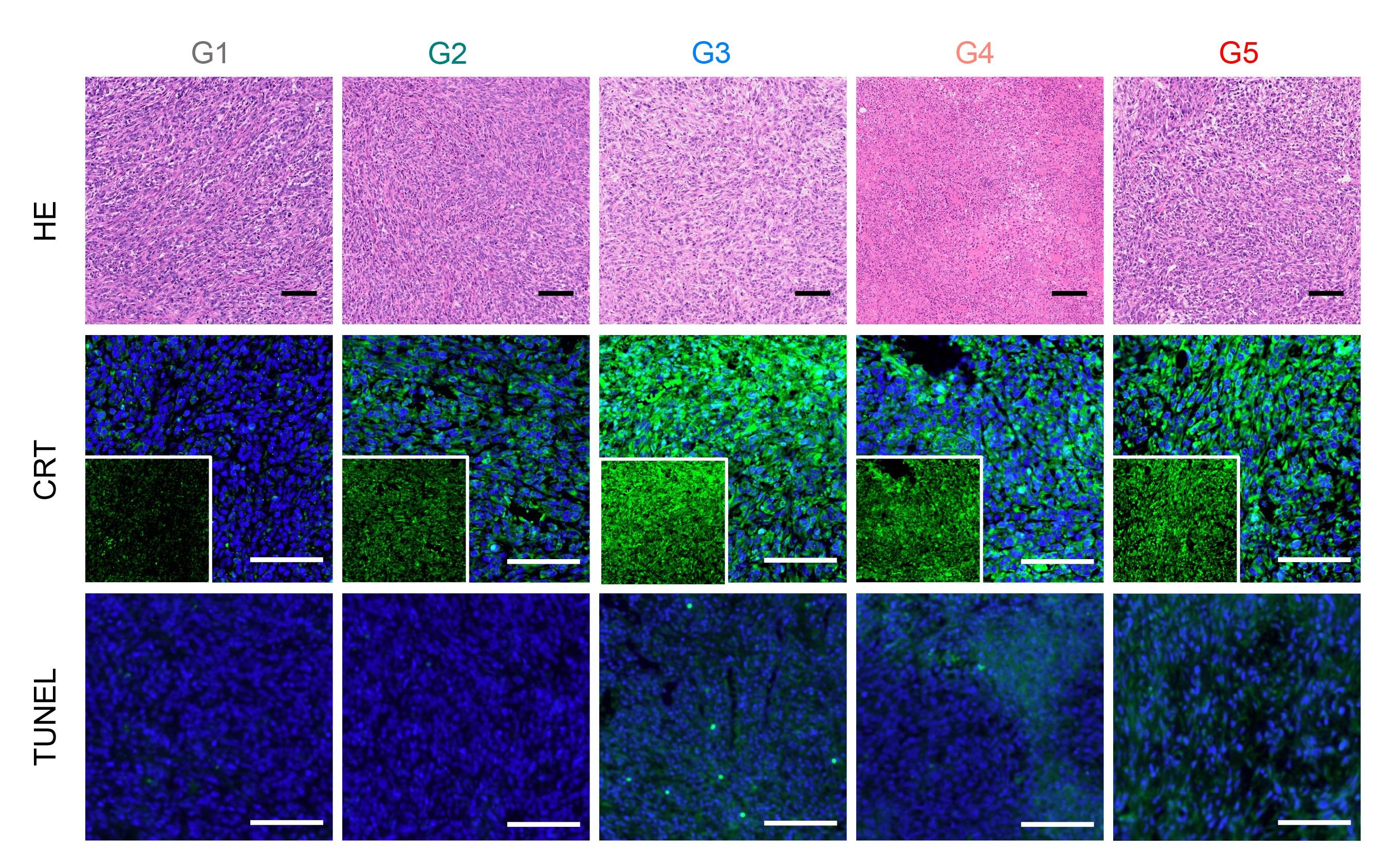


Fgure S12. H&E staining and immunofluorescence staining for CRT and TUNEL in 4T1 tumor tissue sections. Scale bar: 100 μm.


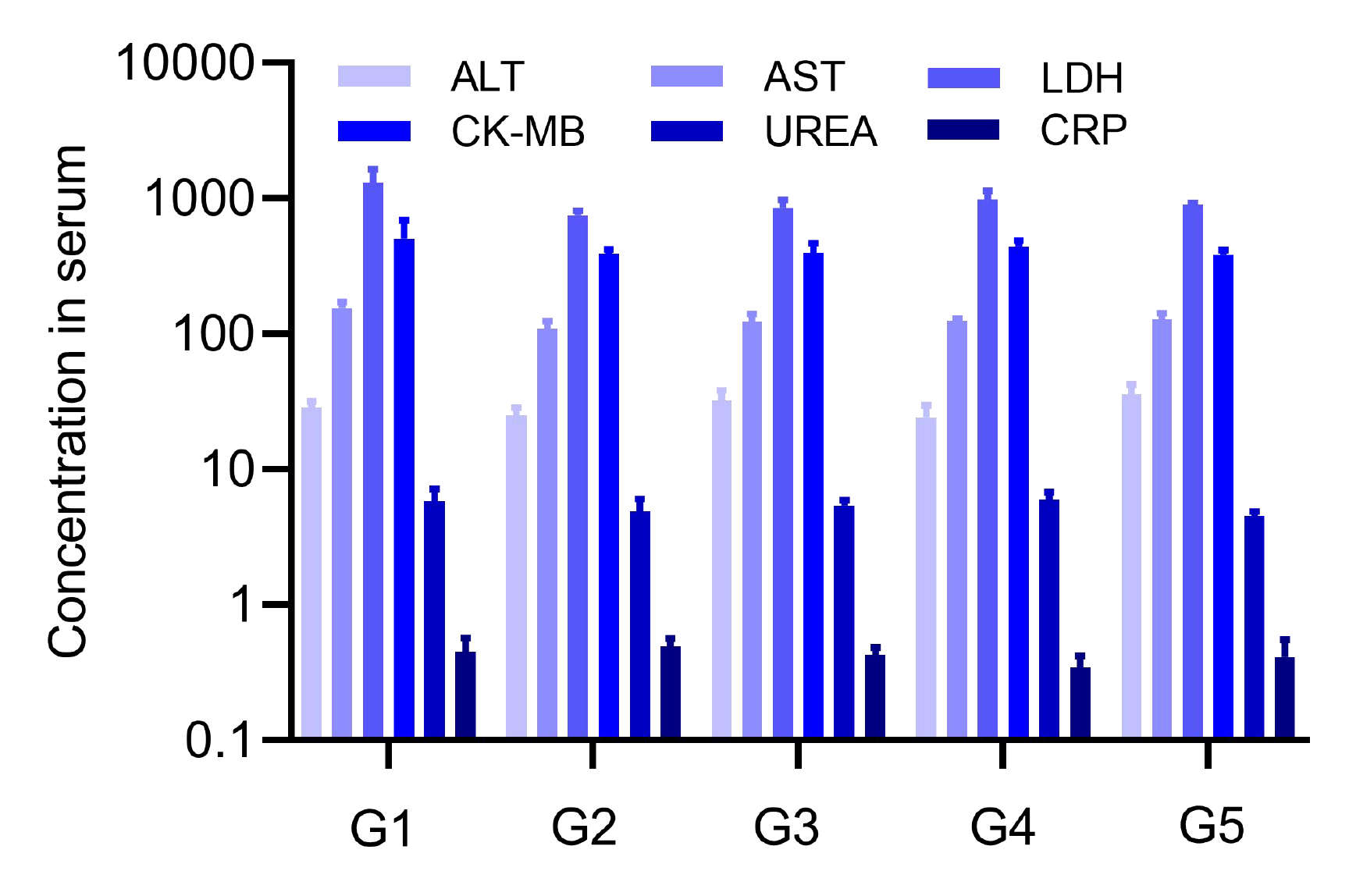


Figure S13. Analysis of alanine aminotransferase (ALT, U L^-1^), aspartate transaminase (AST, U L^-1^), lactate dehydrogenase (LDH, U L^-1^), creatine kinase isoenzyme-MB (CK-MB, U L^-1^), urea nitrogen (UREA, mmol L^-1^), C-reactive protein (CRP, mg L^-1^) in the serum of 4T1 tumor-bearing mice.


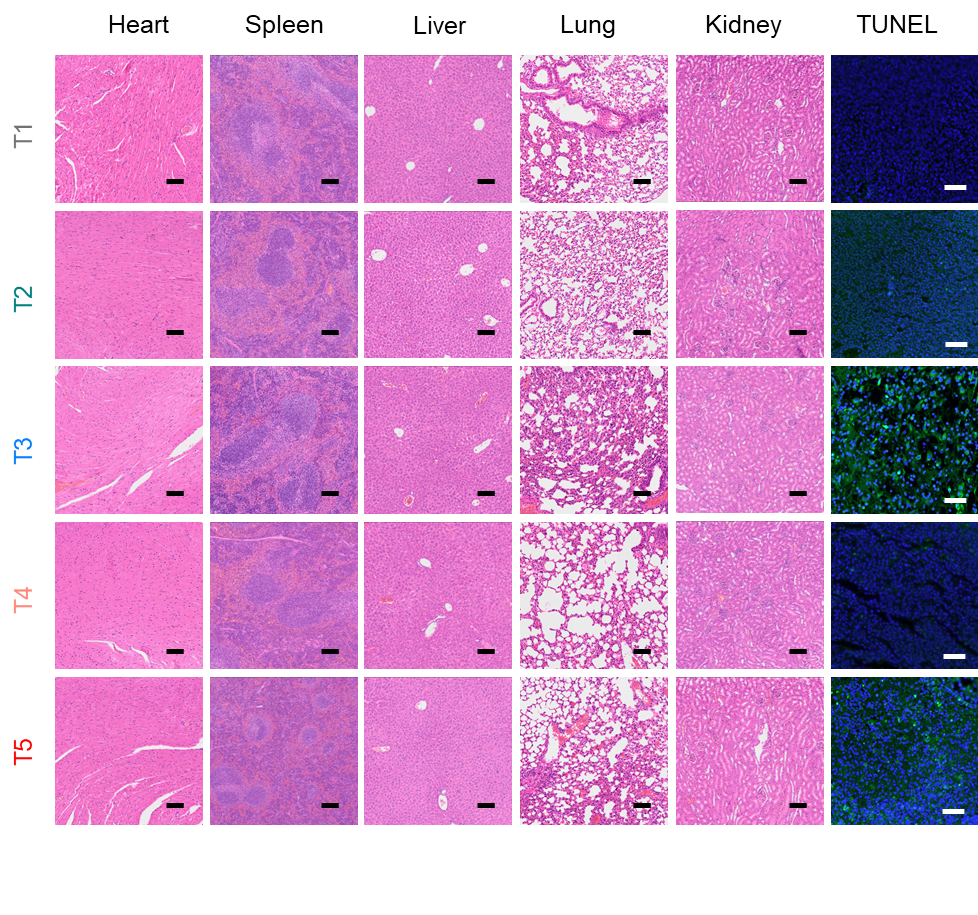


Figure S14. H&E staining of major organs and TUNEL staining of tumors from sacrificed PDX tumor-bearing mice under various treatments. Scale bar: 100 μm.
